# Rubbery organic frameworks (ROFs) toward ultrapermeable CO_2_-selective membranes

**DOI:** 10.1126/sciadv.adq5024

**Published:** 2024-11-13

**Authors:** Marius Sandru, Marie Prache, Thomas Macron, Lidia Căta, Mehmet Göktuğ Ahunbay, May-Britt Hägg, Guillaume Maurin, Mihail Barboiu

**Affiliations:** ^1^ SINTEF Industry, SINTEF AS, NO-7465, Trondheim, Norway.; ^2^ Department of Chemical Engineering, Norwegian University of Science and Technology, 7491 Trondheim, Norway.; ^3^ Institut Européen des Membranes, Adaptive Supramolecular, Nanosystems Group, University of Montpellier, ENSCM, CNRS, Place Eugène Bataillon, CC 047, F-34095, Montpellier, France.; ^4^ Babes-Bolyai University, Supramolecular Organic and Organometallic Chemistry Center (SOOMCC), Cluj-Napoca, 11 Arany Janos str., 400028, Cluj-Napoca, Romania.; ^5^ Institut Charles Gerhardt Montpellier, Univ. Montpellier, CNRS, ENSCM, Place Eugène Bataillon, CC 047, F-34095, Montpellier, France.; ^6^ Department of Chemical Engineering, Istanbul Technical University, Maslak, Istanbul 34469, Turkey.

## Abstract

The capture of CO_2_ is of high interest in our society representing an essential tool to mitigate man-made climate warming. Membrane technology applied for CO_2_ capture offers several advantages in terms of energy savings, simple operation, and easy scale-up. Glassy membranes are associated with low gas permeability that negatively affect on their industrial implementation. Oppositely, rubbery membranes offer high permeability, but their selectivity is low. Here we report rubbery organic frameworks (ROFs) combining the high permeability of soft matrices with the high sieving selectivity of molecular frameworks. The best performing membranes provide a CO_2_/N_2_ selectivity up to 104 with a CO_2_ permeability up to 1000 Barrer, representing relevant performances for industrial implementation. Water vapors have a positive effect on CO_2_ permeability, and the CO_2_/N_2_ selectivity is higher than in dry conditions, as most of CO_2_ gas emissions are present in fully humidified gas streams. The synergetic high permeability/selectivity performances are superior to that observed with current state-of-the-art polymeric membranes.

## Teaser

Elastomeric frameworks show enhanced performances for better processability and upscaling into CO_2_ capture commercial membranes.

## INTRODUCTION

Global warming and greenhouse gas effects are now topics of high interest. They are related to climate change and its negative effects due to human activities. Carbon dioxide, CO_2_, is present in the emissions resulted from power generation by combustion of fossil fuels, steel and cement industries, refineries, and transportation (*1*). An efficient strategy for the reduction of the greenhouse gases emissions is to develop CO_2_ capture technologies adapted to the characteristics of each individual source: CO_2_ content, volume of gas to be treated, location, and gas contaminants (*2*). Among other industrial methods (i.e., absorption in solvents or solid sorbents and cryogenic distillation), the membrane separation technology (*2*–*18*) offers advantages such as energy savings, simple operation, and easy scale-up for main gas separations: (i) CO_2_ versus N_2_ from flue gas (10 to 20% CO_2_, 1 atm, 50°C) (*2*), (ii) CO_2_ versus CH_4_ from natural gas or natural gas sweetening (*3*), and (iii) CO_2_ versus H_2_ for precombustion capture (45% CO_2_, 70 bar, 300°C) (*4*). There are as well several limitations for membrane technology such as (i) the trade-off between CO_2_ permeance and selectivity; (ii) low durability and loss of performances when exposed to water or contaminants present in gas (SO_2_, H_2_S, and hydrocarbons); and (iii) good performance only at high CO_2_ concentrations.

**Fig. 1.**
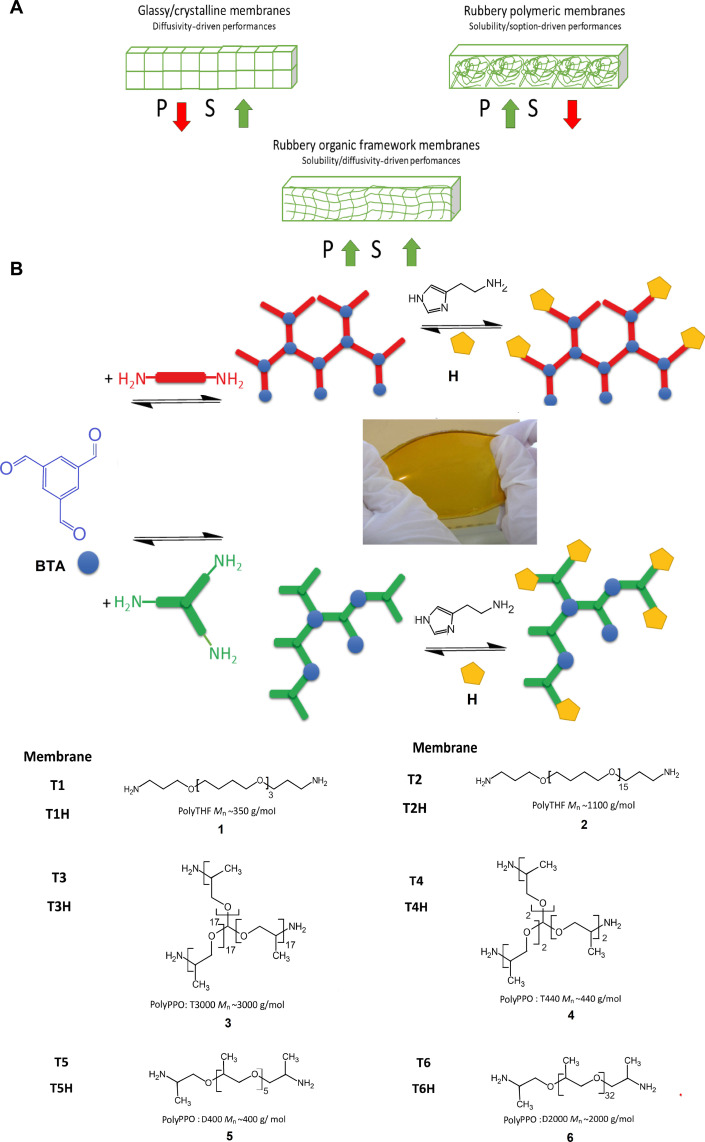
Conceptual and synthetic details of studied ROF membranes. (**A**) Schematic diagram representing the performances of glassy/crystalline, rubbery polymeric, and ROF membranes. *P,* Permeability; *S,* Selectivity. (**B**) Synthesis and schematic representation of chain packing of **T1** to **T6** ROF membranes, combining linear polyTHFs **1** and **2** (red line), star-type **3** and **4** (green star), and linear (red line) **5** and **6** polyPPOs, connected via imine-carbonyl/amine chemistry to BTA core **1** (blue circle). The addition of CO_2_-phylic histamine, **H** heads (yellow) results in the formation of membranes **T1H** to **T6H**. It generates structural diversity of self-standing ROF membrane films with elastomeric behaviors. The photo represents membrane **T6**.

De novo design of membranes for molecular scale gas separations has become an area of expanding interest. Achieving high gas permeability while keeping a reasonable selectivity is usually obtained with membranes for which the gas transport is controlled by the gas diffusivity in glassy materials or by gas solubility in rubbery materials (*5*). The gas diffusivity can be controlled for low permeable glassy polymers and can be improved by using rubbery polymers and increasing the solubility of CO_2_. The gas permeability may be increased by using a rubbery polymer with permeable soft segments (Fig. 1A). Fixed site carrier membranes combine the solution-diffusion mechanism, with a reversible chemical reaction with CO_2_-philic carriers present in the membrane (*6*–*8*). Innovative molecular sieving microporous polymers (PIMs) (*9*–*11*), metal organic frameworks (MOFs) (*12*–*14*), and covalent organic frameworks (COFs) (*15*) have been identified as promising emerging materials for energy-efficient membrane gas separations. Nonetheless, there are many challenges in translating their excellent molecular separation properties into performant scalable and inexpensive industrial-scale membranes and filtration modules needed for effective gas capture. For zeolitic membranes (*16*), film formation becomes limited by local defects and intercrystallite inhomogeneities. Nanocomposite membranes (*13*, *17*) can be prepared to regulate the manner in which MOF and COF fillers and the polymeric matrix can synergistically act. Engineered MOF crystals into polymer matrices may successfully translate their excellent molecular sieving properties into macroscopic mixed matrix membranes, but there are still challenges regarding the membrane area upscaling, module sealing, brittleness, and costly production process (*18*).

Emerging soft and porous crystals (*19*), liquid MOFs (*20*), liquid cages (*21*), and flexible COFs (*22*) with permanent metastable transient porosity arising from local dynamics of framework components have been proposed as continuous phases with enhanced mechanical and thermal properties for improved CO_2_ capture applications (*23*). We postulate earlier by an advanced computational methodology combining quantum and force field molecular simulations (*24*) and experimentally demonstrate in these studies (*25*, *26*) that one of the not yet explored strategies for addressing such scale-up challenges and to achieve improved performance, in terms of both permeability and selectivity, is to use the rubbery organic frameworks (ROFs). The ability to connect rubbery connectors and core centers through reversible bonds controlled at molecular level results in the formation of flexible stable membrane films, combining high gas sorption of permeable rubbery polymers with the selective diffusion of mesh-sieving architectures of selective crystalline polymers (Fig. 1A) (*27*). The key challenge in the construction of such dynamic ROF frameworks is the adaptive formation of gas sieving/taming molecular domains. Elastomeric frameworks can be better processed into membranes compared to Metal Organic Frameworks (MOFs), Zinc Imidazolate Frameworks (ZIFs), or zeolites, preventing the formation of defects. The dynamic connectivity between molecular components results in the formation of homogeneous and robust thin films circumventing the intrinsic poor film formation and brittleness of porous solids. This is achieved by introducing elastomeric domains in a controlled manner while retaining desirable gas-sieving properties of crystalline porous frameworks. We hypothesize that the short distance connectivity in the soft solid state may promote transient intrinsic porosity due to the strong constraints of their three-dimensional (3D) networking at the molecular level, generating adaptive functions for materials. (*22*)

## RESULTS

### Synthesis and structural characterization

Linear or cross-linked macromolecular connectors with nanometric dimensions and connection centers have been used to synthesize a library of ROFs and further fabricate dense self-supported membranes for gas separation. Several building blocks are used as the precursors to prepare the ROFs **BTA**_**2**_**·1**_**3**_, **BTA**_**2**_**·2**_**3**_, **BTA·3**, **BTA·4**, **BTA**_**2**_**·5**_**3**_, and **BTA**_**2**_**·6**_**3**_: benzenetrialdehyde (BTA) bis(3-amino-propyl)polytetrahydro furan and **1**) polytetrahydrofuran (polyTHF) [number-average molecular weight (*M*_n_), ~350 g mol^−1^]; **2**) polyTHF (*M*_n_, ~1100 g mol^−1^); **3**) the star-type glycerol-tris[poly(propyleneglycol)-amine-terminated] ether T3000 (*M*_n_, ~3000 g mol^−1^); **4**) T440 (*M*_n_, ~440 g mol^−1^); **5**) linear bis(3-aminopropyl)-polypropylene glycol D400 (*M*_n_, ~400 g mol^−1^); and **6**) D2000 (*M*_n_, ~2000 g mol^−1^) poly(propylene-oxide) (polyPPOs). Reflux in chloroform overnight followed by evaporation of solvent at 60°C was used to form and cast **T1** to **T6** membrane films (Fig. 1B). A second series of membranes **T1H** to **T6H** were prepared by using the histamine ROFs **BTA·1·H**, **BTA·2·H**, **BTA·3**_**0.66**_**·H**, **BTA·4**_**0.66**_**·H**, **BTA·5·H**, and **BTA·6·H**. These ROFs were used to manufacture a comprehensive library of membranes including a large variety of structural features: (i) The hydrophobic linear polyTHF **1** and **2**, the hydrophilic star-type **3** and **4**, and linear **5** and **6** poly(PPO) have been used to generate the membrane soft phases, allowing a high gas solubility and enhancing the CO_2_ permeability; poly(ethylene-oxide) (PEO) polymers have been used to provide a good balance between CO_2_ solubility and permeation. However, they crystallize (*5*), potentially hindering the permeation of gases. Different methods have been used to decrease the crystallinity of PEO, including the use of low temperature or the use of low molar mass PEO monomers (*28*, *29*). (ii) The presence of methyl groups in PPO prevents the chain packing, suppressing PPO crystallization and increasing the free volume and consequently CO_2_ permeability (*30*). (iii) The ROFs contain a combination of both linear and star-type macromonomers, interconnected via reversible imine bonds, resulting in the formation of frameworks with a certain cross-linking degree that control the free volume at nanometric level. (iv) The bulky connectors have a protecting effect against the hydrolysis of the imine bonds. (v) The histamine, **H** heads are CO_2_-phylic functional subunits, which may increase the water content in the membrane and increase the CO_2_ selectivity of the respective ROFs (*22*–*26*).

The ^1^H nuclear magnetic resonance (NMR) analysis of CDCl_3_ allows easy identification of peaks corresponding to total conversion to imine-substituted **BTA**_**2**_**·1**_**3**_, **BTA**_**2**_**·2**_**3**_, **BTA·3**, **BTA·4**, **BTA**_**2**_**·5**_**3**_, and **BTA**_**2**_**·6**_**3**_ or **BTA·1·H**, **BTA·2·H**, **BTA·3**_**0.66**_**·H**, **BTA·4**_**0.66**_**·H**, **BTA·5·H**, and **BTA·6·H** ROFs. The reported experimental molar compositions have been obtained by the integration of the aromatic and imine protons of N-substituted BTA, using the solvent peak as reference.

The elastomeric behavior for most of the obtained membranes is confirmed by their low glass transition temperatures *T*_g_ ~ −37° to −14°C, with **T1** to **T6** membranes softer than **T1H** to **T6H** ones (table S2). The presence of H-bonding imidazoles from histamine generates crystalline domains in which water clusters can be included. The *T*_g_ values are the lowest for **T3**, **T3H**, **T6**, and **T6H**, indicating an increasing of the free volume of the matrix with the use of poorly packed star-type and longer linear connectors **3** and **6** compared with the compact, well-packed shorter and linear polymer chains of **1**, **2**, and **5** and the highly cross-linked polymer matrix of **4**, leading to glassy **T4** and **T4H** structures. The crystallization temperature *T*_c_, detected for **T2** and **T2H** membranes containing longer polyTHF1100 and high degradation temperatures (320° to 370°C), is probably related to cross-linked frameworks making ROFs suitable for harsh environments or high temperature testing.

The surface morphologies of **T1H** (linear polyTHF, 250 g mol^−1^), **T3H** (star T3000, 3000 g mol^−1^), and **T5** (linear D400, 400 g mol^−1^) membranes were further investigated by atomic force microscopy (AFM; figs. S1 to S3). The AFM images show that these membranes present on surface bubble shapes of 0.2 μm for shorter linear segments in **T1H** and **T5H** and 1 μm for longer cross-linked segments in **T3H**, indicative of high roughness that can be obtained with increasing the mass of the polymers.

Fourier transform infrared (FTIR) spectra demonstrate the formation of polyimines, with the detection of vibration bands at 1450 and 1600 cm^−1^, associated with aromatic moieties conjugated with ─C═N─ bonds for which stretching vibrations are observed at 1640 to 1690 cm^−1^ (fig. S4 and tables S3 and S4). The intensity of the C═N vibration bands strongly depends on the nature of the connectors and is related to the degree of cross-linking of the frameworks. The sharp bands at 1070 and 1150 cm^−1^ attributed to the ─C─O─C─ stretching vibration, as well as the ─CH_2,as_ and ─CH_2,sym_ stretches visible in the range of 2750 to 2957 cm^−1^, are proportional to the number of groups of the PPO chains (tables S3 and S4). Evidence for histamine incorporation was obtained from the vibration band at 1585 cm^−1^ assigned to the C═C stretching of the imidazole. A broad band centered around 3400 cm^−1^ attributed to the O─H stretching vibration of H-bonded water can be observed for some membranes, especially those containing histamine.

Most of the CO_2_ sources such as flue gas are fully humidified, and water absorption in membranes can play an important role in CO_2_ separation, enhancing performances as in the case of fixed site carrier/facilitated transport membranes or decreasing performances by a competitive sorption in glassy polymers or excessive swelling and plasticization (fig. S5). Consequently, water swelling experiments of ROF membranes were performed using controlled fully humidified atmosphere. As expected, the membranes **T1H** to **T6H** containing hydrophilic histamine **H** requires longer equilibrium time to reach steady state (100 hours) and adsorbs almost three to four times more water than the free histamine membranes **T1** to **T6** (60 hours) (table S5 and fig. S6). The imidazole of histamine forms strong H bonds with water, and the membranes **T1H** to **T6H** are expected to absorb more water with longer adsorption time to completely swell the membranes. For histamine **T1H** to **T6H** series, the glassiest polymers absorb more water (**T1H** and **T5H**), and the water uptake decreases with the *T*_g_ decreasing of the more rubbery **T3H** and **T6H** polymers (table S5). For series **T1** to **T6** without histamine, hydrophilic **T1** (linear PEO) had the highest water absorption, and hydrophobic **T6** (linear PPO) had the lowest amount of water. A similar trend is observed, the most rubbery materials **T3** and **T6** have the lowest *T*_g_, absorbing the least amount of water.

### Single and mix gas transport

Single gas N_2_, CH_4_, and CO_2_ permeation experiments have been performed in this order, by using a time lag permeation rig at 5 bar and 25°C (Table 1 and figs. S7 and S8). Mixed gas experiments have also been performed using a gas mixture of 10% CO_2_ to 90% N_2_ at 1.2- and 5-bar feed absolute pressure and 25°C, in dry and humidified conditions (Table 2 and fig. S10).

**Table 1.** Single gas permeation experiments at 5-bar feed pressure and 25°C.

Membrane	Permeability (Barrer)			Selectivity	
	CO_2_	N_2_	CH_4_	CO_2_/N_2_	CO_2_/CH_4_
**T1H**	2.34	0..09	0..17	26	14
**T2H**	196	7	22	30	9
**T3H**	424	15	50	28	8.5
**T5H**	34	13	15	2.5	2.3
**T6H**	517	108	194	4.8	2.7
**T1**	59	1	4	42.5	14
**T2**	250	8	27	31	9
**T3**	711	43	126	17	6
**T4**	119	107	123	1.1	1
**T5**	34	0.81	2.24	42	15
**T6**	895	27	104	33	9

**Table 2.** Mixed gas permeation experiments with dry and 100% RH humidified feed gas 10% CO_2_ in N_2_ at 25°C.

Membrane	CO_2_ permeability (Barrer)				CO_2_/N_2_ selectivity			
	Feed pressure (dry)		Feed pressure (humid)		Feed pressure (dry)		Feed pressure (humid)	
	1.2 bar	5 bar	1.2 bar	5 bar	1.2 bar	5 bar	1.2 bar	5 bar
**T1H**	–	–		13	–	–		3
**T2H**	159	174	115	125	1	5	9	24
**T3H**	282	295	268	267	5	15	5	17
**T5H**	68	108	157	130	11	16	94	104
**T6H**	302	389	318	294	9	7	10	13
**T1**	50	44	55	53	2	6	2	8
**T2**	372	272	418	408	15	50	60	63
**T3**	786	1128	741	726	46	46	62	55
**T4**	55	14	50	49	21	6	20	27
**T5**	59	32	59	40	13	23	47	71
**T6**	1086	898	876	658	31	39	68	54

**Fig. 2.**
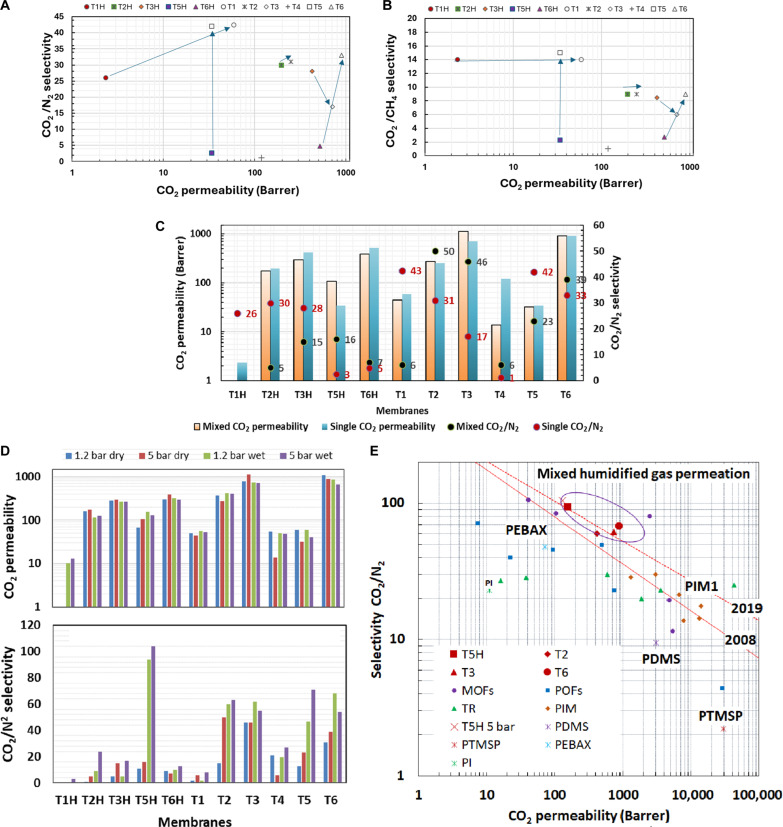
Single and mixed gas experiments. Single gas permeation performances (**A**) CO_2_/N_2_ and (**B**) CO_2_/CH_4_ at 5 bar and 25°C; the arrows show the correlation between membrane with the same composition with (●) and without (○) histamine. (**C**) Comparison of mixed and single gas CO_2_ permeability and CO_2_/N_2_ selectivity at 5 bar and 25°C. (**D**) CO_2_ permeability and CO_2_/N_2_ selectivity of mixed gas experiments at 1.2- and 5-bar feed and 25°C with dry and 100% humidified feed. (**E**) Selectivity-permeability trade-off Robeson graphs; the solid line depicts the 2008 and 2019 upper bounds (*35*). Solid points are showing performances obtained with 10% CO_2_ to 90% N_2_ gas mixture at 1.2 bar, 25°C, humidified feed gas 100% RH. Data for thermal rearranged (TR), fixed site carrier, and PIMs were extracted from literature (*36*). PDMS, polydimethylsiloxane.

As a general trend, the single gas CO_2_ permeabilities and, consequently, the corresponding CO_2_/N_2_ selectivity of the softer (lower *T*_g_) membranes **T1** to **T6** (except for **T3**) are higher by a factor ranging between ×2 and ×16 compared with their equivalent in **T1H** to **T6H** membrane series. This is confirmed and correlated with the CO_2_ sorption data in figs. S12 and S13 and table S7, which implies either a stronger material affinity toward CO_2_ or the presence of a molecular sieving capacity as observed for glassy rigid polymers, a highly unexpected property of a rubbery polymer. When **T1H**, **T3H**, and **T5H** membranes were tested with increased applied feed pressure from 3 to 10 bar, for **T1H** and **T5H**, both CO_2_ permeability and CO_2_/N_2_ or CO_2_/CH_4_ selectivities increased, due to synergetic increase of CO_2_ solubility and sieving properties with pressure. The **T1H** membrane presented a 36% increase and **T5H** membrane a 24% increase in CO_2_/N_2_ selectivity, where CO_2_/CH_4_ selectivity remained constant for **T1H** and increased 21% for **T5H** with pressure (table S6 and fig. S9). It became apparent that, if the length of the polyTHF or polyPPO chains increases (**T2**, **T3**, and **T6** have longer chains versus **T1**, **T4**, and respective **T5**), then the CO_2_, N_2_, and CH_4_ permeabilities increase for both series, while the single gas CO_2_/N_2_ and CO_2_/CH_4_ selectivities increase for **T1H** to **T6H** and decrease for **T1** to **T6** (Table 1 and Fig. 2, A to C). As an example, the single gas CO_2_/N_2_ selectivity increased with chain length by 13% between **T1H** and **T2H** and decreased between **T1** and **T2** by 24% with chain length increase. This can imply that the histamine imidazoles provide more rigidity/compact packing via strong H bonding for **T1H** to **T6H** membranes leading to an increased sieving capacity and higher CO_2_ selectivity. The membrane **T6** has good performance for both CO_2_/N_2_ and CO_2_/CH_4_ separation and presents the best compromise selectivity/permeability. The use of shorter connectors in **T1** and **T5** leads to good selectivity *S*_CO2/N2_ = 41 to 55 and a reasonable CO_2_ permeability (~300 Barrer) for both series. The gas permeability increases with pressure and is correlated with the rubbery state of the polymer. **T2H**, **T3H**, and **T2** show good CO_2_ permeability, however, with a lower selectivity. There is no particular trend observed regarding the N_2_ permeability when comparing the two series.

The permeability and selectivity evaluated from the single gas components are ideal, without taking into account the interaction between gasses and competitive transport in a gas mixture as expected in a gas mixture with CO_2_ concentration below 20% water vapors. Consequently, the performances may change when membranes are tested with mixed gases and in the presence of humidity. The mixed gas permeation experiments performed using 10% CO_2_ in N_2_ gas mixture as feed confirmed these suppositions (Table 2). Two gas feed pressures were used for testing: a low pressure of 1.2 bar close to real flue gas pressure in power generation industry and 5 bar as used for single gas permeation experiments. Testing was performed using both dry and fully humidified [100% relative humidity (RH)] gasses as in the flue gas, breathing, and biogas processes (*2*, *3*).

The CO_2_/N_2_ selectivity substantially varies between mixed and single dry gas tests (Fig. 2C), while no important variation is observed for CO_2_ permeability (except **T4** and **T5H**). All **T1H** to **T6H** membranes present CO_2_/N_2_ selectivity under 30 when tested with mixed dry gas, and all the membranes **T1** to **T6**, except **T4**, have selectivity values higher than 30. Comparing the results at 5 bar, all the **T1** to **T6** membranes, except **T1** and **T5**, presents higher CO_2_/N_2_ selectivity when tested with mixed gases compared to a single dry gas test, supporting the hypothesis of sieving rubbery membranes. For the **T1H** to **T6H** series, the single gas dry test provides better selectivity or similar with the exception of **T5H**.

When membranes were tested with a mixture of gasses, relatively small differences are observed for CO_2_ permeabilities between wet and dry gas conditions: (i) **T1H** to **T6H** membranes (except **T5H**) show a small decrease in permeability, due to competitive water-CO_2_ sorption, as typical for glassy polymers, and (ii) **T1** to **T6** membranes (except **T3** and **T6**) show similar or higher CO_2_ permeabilities under wet conditions. The fact that **T3** and **T6** have similar or lower permeabilities when tested with humidified gas may be correlated with the water sorption where **T3** and **T6** showed the smallest water uptake.

The CO_2_/N_2_ selectivities show a substantial increase when using humidified feed gas for all membranes and feed pressures, the difference being even more pronounced at 5 bar (Fig. 2D). Unexpectedly, for the **T1H** to **T6H** series, the more glassy membranes, despite the CO_2_ permeability being depressed under humid conditions, the CO_2_/N_2_ selectivity is similar or slightly increased. Another obvious trend is that **T1H** to **T6H** membranes, except **T5H**, show lower CO_2_/N_2_ selectivity than **T1** to **T6** membranes under both dry and humid conditions. This is not unexpected because the hydrated imidazoles limit their interactions with the permeant CO_2_ to provide a decreased permeability for **T1H** to **T6H** membranes (*31*). The fact is that the CO_2_ permeability increases with pressure from 1.2 to 5 bar and may indicate a limited contribution of facilitation transport (characterized by higher permeability and selectivity at lower feed pressures) for overall CO_2_ transport mechanism in **T1H** to **T6H** series (*6*–*8*).

An important conclusion based on the results, obtained using humidified and dry mixed gases, is that water vapors have a positive effect both on CO_2_ permeability and especially on CO_2_/N_2_ selectivity except a small decrease of the CO_2_ permeability for the most glassy series **T1H** to **T6H** and **T3** and **T6**. This is beneficial for industrial implementation, as all CO_2_ industrial flue gas, biogas, and natural gas streams are fully humidified and are opposite to the behavior in the presence of humidity of glassy polymer membranes, MOF, and zeolite mixed matrix membranes where the water vapors have a considerable negative effect on membrane performances.

The CO_2_ sorption experiments enabled us to relate the gas transport phenomena with the structure of the membranes (figs. S11 to S13 and table S7). The gas solubility and the diffusion coefficients of CO_2_ are close to the values reported for similar membranes in literature (*32*, *33*). The hysteresis can be observed for most of the **T1H** to **T6H** membranes except the most rubbery ones **T6H** and **T3H**, as a result of the increase of the free volume during the sorption. The more elastic/rubbery **T1** to **T6** membranes do not show a hysteresis, and both sorption and desorption follow Henry’s law. The CO_2_ solubilities obtained through Henry’s law or dual sorption model data fitting are similar for **T1** to **T6** and **T1H** to **T6H** (table S7). This trend is different when compared with water sorption (table S5) as **T1H** to **T6H** membranes adsorb much more water than **T1** to **T6**. Lower CO_2_ permeabilities and CO_2_/N_2_ selectivities for **T1H** to **T6H** series compared to **T1** to **T6** series (except the **T5**/**T5H** pair), under both dry and humid testing conditions (Table 2), indicate that neither the water nor the CO_2_ sorption is the determinant factor for CO_2_ separation. Despite similar CO_2_ solubilities in both series, an order of magnitude difference can be observed for the diffusion coefficients of **T1**/**T1H** and **T5**/**T5H** compared with the rest of the membranes. As in the case of single gas permeation, the separation performances are correlated with structural differences induced by the length of polyTHF and polyPPO chains: Longer chains give higher diffusion. The combination of star shape and long polyPPO chains seems to be beneficial as it provides higher CO_2_ diffusion coefficient. Overall, it can be concluded that diffusivity has the biggest contribution to CO_2_ permeability and is responsible for the observed differences in membrane performances leading to highest CO_2_ permeability for the rubbery membranes **T3H**, **T6H**, **T2**, and **T3**.

### Atomistic-scale molecular simulations

**Fig. 3.**
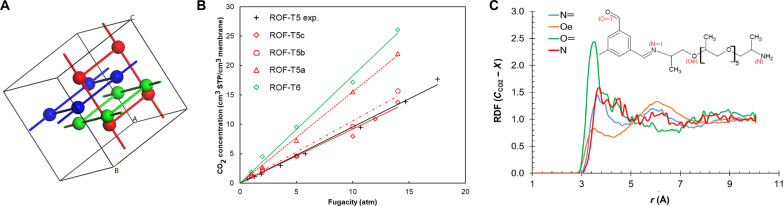
Atomistic-scale simulations. (**A**) Schematic 3D fully interpenetrated cross-linked **T5** ROFs, where the spheres are the BTA centers and the stick are the PPO connectors. (**B**) Simulated CO_2_ sorption isotherms at 298 K in ROF-**T5** and ROF-**T6** models in comparison to experimental data. (**C**) Radial distribution function (RDF) between the carbon atom of the CO_2_ molecule and selected sites of the **T5a** structure. The relevant sites are labeled on the structure fragment in the inset.

To gain further insight on the structure-property relationship of these ROFs, atomistic-scale simulations were carried out for two representative ROFs: **T5** and **T6**, with shorter (polyPPO_5_) and longer (polyPPO_32_) linear connectors, respectively (Fig. 3A). For **T5**, two atomistic models were constructed associated with different degrees of cross-linking, i.e., **T5a** (30%) and **T5b** (100%) varying from a partial to a three imine moieties on BTA center. These atomistic models first identified the effect of the PPO connector length on the rigidity of the ROFs based on the differences of calculated *T*_g_ values of **T5** and **T6**, which are estimated as −12°C for **T5a**, 8°C for **T5b**, and −30°C for **T6**, through the simulated cooling via NPT and NVT–molecular dynamic (MD) simulations describing the system at constant particle number (*N*), pressure (*P*), volume (*V*), and temperature (*T*) (figs. S14 and S15). When compared to the experimental data (−28° and −56°C, respectively), the simulation reproduced the effect of the structural differences on the *T*_g_ values consistently. It should be noted that a faster cooling rate in molecular simulations leads to overprediction of *T*_g_ by ~30°C, according to the Williams-Landel-Ferry model (*32*).

Figure 3B reports the calculated CO_2_ sorption isotherms at 298 K via a strategy integrating grand canonical Monte Carlo simulations and NPT- and NVT-ensemble MD simulations (more details in the Supplementary Materials) for the two **T5** models and compared them to the experimental sorption isotherm collected for the **T5** membrane. The cross-linking of **T5** implies, as expected, a reduction in the CO_2_ sorption capacity. The resulting Henry’s constants are 1.353 and 0.921 cm^3^/atm for **T5a** and **T5b**, respectively, with respect to the experimental value of 1.421 cm^3^/atm. The comparison between experimental and simulated sorption isotherms suggests that the polymer **T5** is therefore partially cross-linked. Radial distribution function analyses reported in Fig. 3C for different CO_2_/**T5** atom pairs shows that the CO_2_ affinity exhibited by **T5** is associated to preferential interactions between the carbon atom of CO_2_ and the oxygen atom of the carbonyl group. The simulated CO_2_ sorption isotherm in **T6** is also shown in Fig. 3B. This simulation shows that **T6** has a higher CO_2_ sorption capacity due to its longer PPO chain, which makes it more flexible and exhibits a greater swelling tendency. In addition, Henry’s constant of 1.825 cm^3^/atm is higher than the values obtained for both **T5** models, in line with a stronger interaction between CO_2_ and **T6**.

MD simulations performed at 298 K and at the equilibrium loadings at 5 bar, in line with the experimental protocols, further revealed that CO_2_ diffuses faster in **T6** (*D*_s,CO2_ = 5.47 × 10^−6^ cm^2^/s) than in both **T5** models (*D*_s,CO2_ = 0.133 × 10^−6^ cm^2^/s for T5a and *D*_s,CO2_ = 0.611 × 10^−6^ cm^2^/s and for T5b), in line with the experimental CO_2_ diffusivity data reported in table S7. This trend is associated with the more pronounced chain mobility of **T6** that enables generation of free volume and diffusion pathway for the gas. The separation performances of **T5** and **T6** were further evaluated in terms of CO_2_ permeability and CO_2_/N_2_ and CO_2_/CH_4_ selectivity based on single components and binary mixture conditions. For a better insight of the separation mechanism of these polymers, it is of interest to break down the permselectivity (α_P_) into solubility selectivity (α_S_) and diffusion selectivity (α_D_), based on the solution-diffusion model. Moreover, comparison of ideal and mixed gas solubility selectivities allows identifying the presence of any competitive sorption phenomenon. Table 3 summarizes the calculated single gas and binary mixture separation properties for CO_2_/N_2_ and CO_2_/CH_4_ gas pairs in **T6** at 298 K and 5 bar that are in excellent agreement with the experimental data presented as well in this table. For binary mixtures, CO_2_:N_2_ ratio was set to 10:90 in line with the experimental work, where as a 50:50 mixture was assumed for the CO_2_/CH_4_ pair. These simulations show that gas diffusivity increases with the length of the PPO chains. As typical with rubbery polymers (*33*, *34*), the membranes are not selective in terms of diffusivity, and the CO_2_/N_2_ separation in **T6** is governed primarily by sorption selectivity. High diffusivity and high sorption selectivity result in high permselectivity, confirming the experimental observation that **T6** is a promising candidate for CO_2_/N_2_ separation. On the other hand, the relatively poorer performance of this polymer in CO_2_/CH_4_ separation is due to lower solubility selectivity.

**Table 3.** Simulated single gas transport properties of T6 at 298 K.

	Simulation						Experimental	
	*S*_i_ (cm^3^/cm^3^)	*D*_s,I_ (10^−6^ cm^2^/s)	*P*_i_ (Barrer)	α_S_	α_D_	α_P_	*P*_i_ (Barrer)	α_P_
CO_2_	9.75	5.47	1404				898	
N_2_	0.0902	4.28	51	21.6*	1.28*	27.7*	27	33*
CH_4_	1.41	2.89	111	6.95†	1.96†	12.7†	104	8.6†

*CO_2_/N_2_ selectivity.

†CO_2_/CH_4_ selectivity.

## DISCUSSION

ROF membranes reported here present high CO_2_ permeabilities and unexpectedly high selectivity for a rubbery-based membrane under various gas transport conditions, including changes in pressure, humidity, and single and mixed gas transport experiments. This suggests that the ROFs designed from low molecular components are intrinsically promoting the preferential selective transport and separation of CO_2_ presenting higher selectivity (CO_2_/N_2_, 50 to 100) compared to classical elastomeric polymers.

In addition, when compared to glassy polymers and MOF or zeolite mixed matrix membranes (*35*–*37*), the ROF membranes are not negatively affected by the water sorption, water vapors having a positive effect on CO_2_/N_2_ selectivity. The high CO_2_ permeabilities (500 to 1000 Barrer) can be explained by the rubbery state of the polymer. Hysteresis phenomena were observed during CO_2_ sorption experiments and corroborated with the generation of a transient high free volume into ROF matrix resulting in increased diffusivity. High CO_2_ permeability is related as well to high affinity of CO_2_ to the PPO chains in **T3** and **T6** due to chemical interactions, pointing toward the possibility of a facilitated transport contribution to the overall transport mechanism. Robeson diagram for CO_2_/N_2_ separation, with both 2008 and 2019 proposed upper bonds, shows that three membranes of this study, **T3**, **T6**, and **T5H**, are above the 2019 upper bond (Fig. 2E) (*35*, *38*). This implies excellent permeability performances when compared with other (i) polymeric rubbery poly(dimethylsiloxane)(PDMS) or poly(1-trimethylsilyl-1-propyne) (PTMSP) or glassy polyimide (PI) or PEBAX membranes or (ii) porous polymer of intrinsic microporosity (PIM) and TR polymeric or mixed matrix MOF and POF membranes showing synergetic high permeabilities and selectivity. It also suggests an excellent structural homogeneity, a necessary requisite to manufacture seamless membranes without defects. The excellent film formation processability and stability of ROFs make these membranes excellent candidates for upscaling and implementation in industrial CO_2_ separation processes.

## MATERIALS AND METHODS

### Chemicals

All reagents were obtained from Sigma-Aldrich and used without further purification. All organic solutions were routinely dried by using sodium sulfate (Na_2_SO_4_).

### General procedure for the synthesis of ROF membrane films

One mole of the BTA and 0.66 mol of difunctional connectors **1**, **2**, **5**, and **6** or 1 mol of trifunctional connectors **3** and **4** were solubilized in 80 ml of methanol or chloroform. The reaction was vigorously stirred overnight at reflux. The mixture was concentrated to almost the half volume, 40 ml. Then, 7 ml of solution was poured into a Teflon dish (6 cm in diameter) and slowly dried at 25°C for 4 days to give yellow films. In the final step, the films were heated at 70°C in an oven for two more days. ^1^H NMR spectra were recorded on an ARX 300 MHz Bruker spectrometer in CDCl_3_ using the residual solvent peak as reference.

### Characterization of the membranes

The membranes **T1H** to **T6H** and **T1** to **T6 **have been characterized by differential scanning calorimetry, FTIR, AFM, gas permeation, and gas and water sorption. A summary of the experiments made is presented in table S1 (*39*).

### Swelling experiments

The membranes in this study are intended to be used for CO_2_ capture from flue gas, natural gas, and biogas. For all these applications, the gas is fully humidified. In addition, water plays an important role in material durability, as membranes can excessively swell and degrade. It is known that water can play a favorable role in CO_2_ transport as in the case of facilitate transport membranes, and here, it can interact with donor-acceptor H-bonding groups and easily solvates acidic gases as CO_2_. It can therefore be useful to know to which extent the polymer is able to uptake water. To measure the amount of water absorbed by the membranes, swelling experiments have been performed in a controlled water vapor fully saturated atmosphere. The membranes have been dried for 90 hours in a vacuum oven at 50°C, and dried weight was measured and recorded. The membranes were installed in a “humidity box” together with an open recipient filled with water. The box was covered with aluminum foil to guarantee a 100% humidity atmosphere (more details in the Supplementary Materials).

### Gas permeation experiments

The membranes investigated were prepared as self-standing films and were supported on filter paper for facile manipulation during testing. The membranes with filter paper were mounted in a sandwich mode, between two discs of aluminum tape. For the most flexible membranes, which easily deform when tighten in the testing membrane cell, smaller membrane areas were used. The diameter of the hole in aluminum was at least 4 mm smaller than the membrane diameter. A rubber O ring is used on top to hold the membrane sandwich on top of a stainless-steel sinter filter in the bottom of the cell. Very often, gas transport properties of new membrane materials are tested by single gas permeation using the time-lag method, relatively high feed pressure, and hard vacuum on permeate side (below 10 mbar). Although in real life, a mixture is always present, often containing water vapors (fully water saturated), with the interest component to be removed in low concentration present (i.e., CO_2_, 0.4% in air to 15% in flue gas from coal fired power plants), single gas permeation can provide valuable indications about the gas transport mechanism, gas solubilities, and diffusivities. In our experiments, after an overnight evacuation, a feed pressure of 5 bar of nitrogen was applied during approximately 2 hours, and permeation was measured. After 4 hours of evacuation, 5 bar of methane was applied, again followed by 4 hours of evacuation before the application of 5 bar of carbon dioxide. For the purpose of screening, feed pressure was maintained constant at 5 bar and the temperature at 25°C. In a second step, for some selected membranes, the pressure influence on gas permeability was investigated additionally at 3-, 8-, and 10-bar feed pressure.

### Single gas permeation experiments

The single gas permeation setup is presented in fig. S8. The feed gas is provided from gas cylinders, and a pressure sensor enables the control and check the applied pressure. Vacuum is applied on the permeate side of the membrane using a vacuum pump. After placing the cell in the gas permeation setup, a certain evacuation time was needed to be sure that all the gases adsorbed on the membrane and on the tubes of the apparatus were desorbed. For this, all the valves were opened, and the vacuum pump was running. An overnight evacuation was performed, at the installation of the cell. A leakage test has been performed for the permeate side: After evacuation, all the valves were closed, and the pressure in the permeate side was followed. Then, the slope of the pressure versus time gives the permeate side leakage rate. Each membrane was tested at 5 bar with different gases. The gases used were nitrogen (N_2_), methane (CH_4_), and carbon dioxide (CO_2_), in this order, to avoid modifications of the membrane due to the strongest adsorbing gases. The pressure was set in the feed side using the first pressure sensor (between V1 and V3), the valve V6 was opened after that, and valves V10, V11, and V12 have been closed. The second pressure sensor (in the permeate side before V11) enabled to follow the increasing pressure. Between two gases, the whole system was evacuated, for at least twice the time of exposure to gas.

### Mixed gas permeation experiments

The membranes used were the same membranes used for the single gas experiments. Each membrane was tested with a 10% CO_2_ to 90% N_2_ at 1.2 and 5 bar in both dry and wet conditions. The dry experiments were run first, and then exposure to wet gases was performed overnight before taking measurements. The sweep gas used was pure methane or pure helium at a flow of 5.6 ml/min and an absolute pressure of 1.02 bar. A leakage test was run with pure CO_2_ or CH_4_ to measure the nitrogen amount leaking into the setup during the experiments. The permeate gas was sent to the chromatograph, and the permeate composition was recorded at steady state. A schematic representation of the testing setup used for mixed gas permeation experiments is presented in fig. S10. A pressure indicator enables the measurement of the feed pressure. The retentate gas flow rate is measured with bubble flow meter. The sweep gas pressure is set to atmospheric pressure, and the flow can be adjusted and recorded via a flow meter or a flow controller. The permeate gas is sent to a gas chromatograph to analyze its composition. Two different configurations are possible: using dry gas or humidified gas. In case of humidified, wet gas, both feed and sweep gases follow are passed through a diffuser in a metallic cylinder filled with water. The membranes used were the same membranes used for the single gas experiments. Each membrane was tested with a 10% CO_2_ to 90% N_2_ at 1.2 and 5 bar in both dry and wet conditions. The dry experiments were run first, and then exposure to wet gases was performed overnight before taking measurements. The sweep gas used was pure methane or pure helium at a flow of 5.6 ml/min and an absolute pressure of 1.02 bar. A leakage test was run with pure CO_2_ or CH_4_ to measure the nitrogen amount leaking into the setup during the experiments. The permeate gas was sent to the chromatograph, and the permeate composition was recorded at steady state (more details in the Supplementary Materials).

### Gas sorption experiments

A gravimetric method was used to characterize the sorption of the different gases in the membranes. The experiments were conducted using a Rubotherm magnetic suspension balance. The specificity of this instrument is that the sample and the holder are not directly linked to the balance, and so very extreme conditions of pressure and temperature can be applied. The holder is maintained suspended by a permanent magnet. The system is presented in fig. S11. The system enables the measurement of the variation of weight of a sample holder and the sample under different conditions of pressure and/or temperature. This variation can be related to the specific gas uptake. The plot of specific gas uptake at one given temperature versus pressure is called sorption isotherm of the gas in the membrane. To avoid perturbations due to non-ideality, the fugacity was used instead of the pressure. Before all experiments, the weight of the holder and the volume of the holder are measured. A “blank” measurement is run using different pressures of helium, which is considered as a non-adsorbent gas, which are applied to the holder only without sample (more details in the Supplementary Materials).

### Atomistic simulations

The nonbonded interactions between the ROF atoms were represented by the Transferable Potentials for Phase Equilibria-United Atom (TraPPE-UA) force field (*40*), while bonded interactions were taken from the General Amber Force Field (GAFF) (*41*). A 14-Å cutoff was applied for both Lennard-Jones (LJ) and electrostatic interactions, accompanied by tail corrections for LJ interactions and Ewald correction for the long-range electrostatic interactions (*42*). Partial electrostatic charges of the polymer atoms were estimated by density functional theory calculations using the Dmol3 tool of the Materials Studios 5.5 simulation package (BIOVIA Dassault Systems, San Diego) based on the repeat unit of ROF-T5. To represent the permeant molecules, the Elementary Physical Model EPM2 force field was adopted for CO_2_ (*43*), whereas methane was represented as spherical united atom (*40*). For N_2_, the three-site model was used with a dummy charge at the center of mass of the molecule (*44*). For the ROF-type polymers with short PPO chains, such as ROF-T5, the representation of the cross-linking between the carbonyl group of the BTA and the amine group of the PPO could be important, since the degree cross-linking controls the chain mobility of the polymers and their sorption capacities. To understand the extent of this effect, we considered two different cross-linking schemes for the ROF-T5: (i) hyperbranching and (ii) fully cross-linking. For the hyperbranched ROF-T5 model, a repeat unit containing trialdehyde and PPO groups was used to grow a hyperbranched copolymer of ~31 kDa using the polymer builder tool of the Materials Studio software package. This procedure led to a cross-linking degree of 30%, which was defined on the basis of the ratio of NH_2_ to N═O groups. Two of these chains were used to construct a cubic simulation cell using the Amorphous Cell module of the software package. The matrix was then equilibrated using a series of NVT- and NPT-MD simulations. The fully, 100%, cross-linked ROF-T5 model was constructed by inserting three repeat units orthogonally leading to a 3D network as represented by the cartoon in Fig. 3A, which forms a unit cell. The unit cell was then extended in all three dimensions by creating a 2 by 2 by 2 super cell. The resulting supercell underwent a series of NVT- and NPT-MD simulations. For the ROF-T6 polymer, only an uncross-linked model was constructed because of the higher chain length of the PPO, which makes the effect of the cross-linking negligible. Two hyperbranched T6 chains of ~30 kDa were constructed and terminated by NH_2_ groups. These chains were used to obtain a simulation box with a final density of 1.05 g/cm^3^ after the equilibration procedure. For polymers, the *T*_g_ is an important descriptor of the chain flexibility. As an initial validation of the polymer models, the glass transition temperatures (*T*_g_s) of the ROF-T5 and ROF-T6 polymers were estimated by calculating the specific volume (*v*_i_) of each polymer over the temperature range (160 to 360 K) using NPT-MD simulations. Starting from 360 K, each polymer is cooled down from with 20 K decrements, which mimicked the experimental cooling procedure. The temperature at which the slope of the “*v*_i_ versus *T*” curve changed was identified as the *T*_g_ for the polymer. The open-source Large-scale Atomic/Molecular Massively Parallel Simulator (LAMMPS) software was used in all molecular simulations (Monte Carlo and MD). All postprocessing analyses were carried out by using either the modules available in LAMMPS or in-house codes unless otherwise stated (more details in the Supplementary Materials).

## Supplementary Materials

### The PDF file includes:

Supplementary Text Tables S1 to S8 Figs. S1 to S15 Legends for data S1 and S2 References
