# Supplementary material for: Rubbery organic frameworks (ROFs) toward ultrapermeable CO2-selective membranes
Source: Sci Adv. 2024 Nov 13;10(46):eadq5024. doi: 10.1126/sciadv.adq5024 (PMC11559614; doi:10.1126/sciadv.adq5024)
Supplement: Supplementary file 1 — Supplementary Text Tables S1 to S8 Figs. S1 to S15 Legends for data S1 and S2 References [file sciadv.adq5024_sm.pdf]

Science Advances
AAAS

Supplementary Materials for

Rubbery organic frameworks (ROFs) toward ultrapermeable CO_2-selective membranes

Marius Sandru *et al.*

Corresponding author: Mihail Barboiu, mihail-dumitru.barboiu@umontpellier.fr

Sci. Adv. **10**, eadq5024 (2024)
DOI: 10.1126/sciadv.adq5024

The PDF file includes:

Supplementary Text
Tables S1 to S8
Figs. S1 to S15
Legends for data S1 and S2
References

Other Supplementary Material for this manuscript includes the following:

Data S1 and S2

1. General information of carbon capture technologies

In 1992 at the United Nations Framework Convention against Climate Change (UNFCC) the UN countries recognized that "*the climate system is a shared resource whose stability can be affected by industrial and other emissions of carbon dioxide and other greenhouse gases.* In 1997, a decision was taken to take commitments in order to reduce the greenhouse gas emissions through the Kyoto Protocol. which together will follow up meetings have been and are still highlighted by most of the media in the world, and thus the public is fully aware about the problem of global warming.
With the increased industrialization the proportion of CO_2 has dramatically increased during the last years. The capture of CO_2 in fossil fuel power plants and energy intensive industries such as steel, oil, transport, petrochemical industry, cement industry has been extensively studied and three CO_2 capture paths with different issues, advantages and disadvantages emerged:

- Post-combustion capture: treatment of various flue gas sources, meaning that the separation is mainly between nitrogen and carbon dioxide. The partial pressure of CO_2 is low but the process can be easily adapted to existing plants.
- Pre-combustion capture: after gasification of the feed gas, a gas-rich in hydrogen and carbon dioxide is obtained. The separation occurs between H_2 and CO_2. The driving force is high as the partial gases pressure is high.
- Oxy-combustion capture: The combustion occurs using an oxygen rich gas so the exhaust gas obtained is rich in carbon dioxide. Once again, the driving force is high but the process is energy consuming to produce oxygen rich feed gas.

For the captured CO_2 storage, two main pathways are investigated: geological and maritime storage. Another place where carbon dioxide capture is required is the production of liquefied natural gas (LNG) from natural gas. Natural gas is mainly composed of methane with many other gases such as CO_2, SO_2 and other alkanes. Those impurities can freeze at the storage and transport conditions of methane (-162 °C and 0.1 bar) and thus need to be removed before liquefaction. This separation involves then methane and carbon dioxide.
There are several processes to remove CO_2 from a gas mixture, among these, two processes received considerable attention:

- Absorption process: the gas flows in a counter-current configuration with a chemical or physical solvent and one of the components has better affinity for the solvent (or can react with the chemical solvent) and is removed. This process needs a step of regeneration of the solvent, a step which is energy consuming..

- Membrane process: Membranes can be prepared from organic, inorganic or mixed materials. The separation occurs due to one component having better affinity for the membrane and is able to go through more easily than the others. This technology requires in general less energy than absorption and does not make use of chemicals. However, a main challenge is to develop materials with both high CO_2 permeability and CO_2 selectivity which are sufficiently robust, cost-effective and can be facile upscaled for large scale industrial applications.

Our suggested approach, ROF membranes, are soft, rubbery polymers that mimic the high selective properties of inorganic based materials MOF' s COF' s or zeolites without their backdowns, poor processability, brittleness, and relatively high cost

2. Synthesis of the ROF Membranes

Table S1. Summary of the experiments.

Membrane	Thickness (μm)	Gas permeation	Gas Sorption	DSC	FTIR	AFM	Comments
**T1H**	152	Yes	Yes	Yes	Yes	Yes	
**T2H**	177..7	Yes	Yes	Yes	Yes	-	
**T3H**	150	Yes	-	Yes	Yes	Yes	
**T4H**	214.3	-	-	Yes	Yes	-	Membrane too brittle: broken during experiment
**T5H**	170	Yes	Yes	Yes	Yes	-	
**T6H**	102.9	Yes	yes	Yes	Yes	-	
**T1**	281.25	Yes	Yes	Yes	Yes	-	
**T2**	192.19	Yes	Yes	Yes	Yes	-	
**T3**	287.5	Yes	Yes	Yes	Yes	-	
**T4**	181.43	Yes	-	Yes	Yes	-	Sorption experiment missing
**T5**	225	Yes	Yes	Yes	Yes	Yes	
**T6**	517.14	Yes	-	Yes	Yes	-	Sorption experiment missing

3.1. Differential Scanning Calorimetry- DSC

The DSC analyses were performed applying different cycles of heating and cooling (Table S2):

- Heating from -70°C to 120 °C and cooling again until -70°C
- If appropriated, cycle around one interesting temperature (Tg, Tm)
- Heating from -70°C to 250°C and cooling again until -70°C
- Heating from -70°C to 400 °C and cooling again until -70°C

Several different types of peaks were identified and interpreted:

- First order peak: the corresponding characteristic temperature is at the top of the peak.
  - melting: endothermic peak in heating curve
  - crystallization: exothermic peak in heating or cooling curves
- Second order peak: the characteristic temperature is the temperature of the inflexion point
  - glass transition: exothermic transition in heating and in cooling curves.

Thermogravimetric analysis (Hi-Res TGA 2950, TA Instruments, Nitrogen, 50-600 °C at 10°C/min) and Differential Scanning Calorimetry (DSC 2920 Modulated, TA Instruments) were used to evaluate thermal stability of the dynameric materials.

Table S2. DSC analysis of T1H-T6H and T1-T6 as measured at 10°C/min under nitrogen.

Membrane	Tg (°C)	Tc (°C)	Tm (°C)	Td (°C)	Room temperature appearance
**T1H**					Glassy, brittle
**T2H**		-34	1,8		Rubbery, soft
**T3H**	-53			337	Rubbery, soft
**T4H**	66				Glassy, very brittle
**T5H**	-4				Glassy
**T6H**	-56			352	Rubbery
**T1**	-37		375		Rubbery
**T2**		-25	1	372	Rubbery
**T3**	-55			357	Rubbery
**T4**	38				Glassy
**T5**	-14		348		Rubbery, soft
**T6**	-59			321	Rubbery, soft

❖ **Membrane T1H:** T1H is a glassy polymer and the fact that no characteristic temperatures can be found by DSC shows that it is an amorphous or a semicrystalline rubbery polymer.

❖ **Membrane T2H:** T2H has the visual appearance of a rubbery polymer. The DSC results show that no glass transition appears (maybe it is below -70°C) and a melting peak is seen at 1.8°C. This could correspond to the melting of the crystallites of the soft segment of the copolymer (the PEO part). The exothermic peak around -35°C in the heating curve is related to the dynamic state of the copolymer. On the cooling part, the crystallization of PEO happens around -37°C. On the second cycle, the melting temperature is a little higher.

❖ **Membrane T3H:** T3H has a visual appearance of a rubbery polymer and the glass transition temperature found by DSC, -52°C, is coherent with the fact that at room temperature the polymer is completely in the rubbery domain. This can also be deduced from the structure: T3H has a structure which prevents tight chain packing with very long flexible chains made of 17 ether groups. Those groups are propylene oxide groups, and those polymers are known to be amorphous at room temperature. This means that, despite its star shape, it is a flexible polymer and consequently a rubbery one. Consequently, the free volume of this polymer should be high and the gass diffusivity is expected to be also high.

❖ **Membrane T4H:** T4H has the same star shape structure as T3H but with very shorter PPO chains. The polymer chain flexibility is lowered, and the chain becomes more rigid due to presence of benzene and histamine groups: the polymer is glassy. This is confirmed as well by the the DSC analysis: T4H has a high glass transition temperature of 66°C and at room temperature it is still in the glassy state.

❖ **Membrane T5H:** From the DSC results we could infer that T5H is a rubbery polymer because of its low Tg (-6°C) whereas from the visual observation appears rather glassy. The structure of T5H could lead also to the interpretation of a quite glassy polymer.

❖ **Membrane T6H:** The very low Tg obtained by DSC (-55°C) is coherent with the visual observation of a rubbery polymer. This is also obvious from the chemical structure, with long PPO groups of the T6H.

❖ **Membrane T1:** The low Tg (-37°C) measured is coherent with the rubbery state of the membrane at room temperature. Therefore, the absence of histamine groups has a strong influence because no glass transition temperature was visible for T1.

❖ **Membrane T2:** As for T2, no glass transition temperature is observed although the polymer is rubbery, it might be under -70°C. There are also strong exothermic and endothermic peaks: a first exothermic peak at -25 °C, most probably due to phase transition of polyTHF chains, a melting peak around 1°C and, on the cooling curve, a crystallization peak around -39°C. T2 without histamine and T2 seem much closer than T1H and T1. As the polyether chains are much longer for T2 than for T1, the effect of the histamine group can be diluted, less important.

❖ **Membrane T3:** The low Tg (-55°C) of T3 is in accordance with the visual aspect at room temperature and close to the Tg observed for the membrane containing histamine. Once again, the long polyether chain might reduce the effect of histamine groups on the packing and consequently on the characteristic temperatures observed. As for T3 with histamine, the polymer is degraded before 400^{0}C but at a higher temperature (357°C instead of 337 °C).

❖ **Membrane T4:** The T4 is the only one from this series which has a stiff, glassy appearance at room temperature and this is confirmed by DSC experiments: the glass transition temperature (38°C) is higher than room temperature. However, this temperature is lower than the one of T4H (66°C). The polyether chains are short, and the density of histamine groups is therefore important. Their effect on the properties of the polymers is important.

❖ **Membrane T5:** The T5 rubbery polymer has a low Tg (-14°C), lower than T5H (-6°C). The polyether chain length (5 units) is average between the shortest (for T4: 2 units) and the longest (for T6: 32 units) and the effects of the histamine groups are more pronounced.

❖ **Membrane T6:** T6 membranes present long PPO chains (32 units) and the effect of histamine groups is not very pronounced on the glass transition temperatures (-57°C for (T6) versus -56°C (T6H) or degradation temperatures (321°C versus 352°C). The membrane appearance is rubbery which is coherent with the DSC characteristic temperatures.

3.2. Atomic Force Microscopy (AFM)

The AFM characterizations were made using a Veeco Multimode V in tapping mode with a J-scanner from NTNU NanoLAB https://www.ntnu.edu/nano/english

❖ **T1H:** The relief of the surface of T1H it is observed based on a color gradient representation: from the deepest indentation to the highest protuberance the colors gradient change from the darkest to the lightest shades. It can be noticed that the surface is not flat and presents many irregularities on the the 10 μm^2 picture. The parallel lines which can be seen on the 10μm^2 picture seem to be artefacts from the AFM machine but the lines on the 1μm^2 picture could show some order of the chains of the polymer. Indeed, the lines seem to follow the relief.

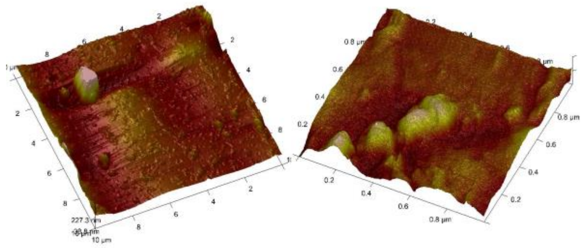

Figure S1. AFM pictures of T1H (with histamine): Left, area of 10µm^2; right,area of 1µm^2.

- **T3H:** The surface of T3H show a different aspect than T1H one: some dome shape (bubbles) features and cracks are visible on the 10 µm^2 picture. The bubbles might indicate regions of the hard segments. On the 1 µm^2 picture, some structural organization can be seen with the curves at the bottom of the picture. If the main parallel lines are due to some artefact, the grain can be due to the structure of the polymer.

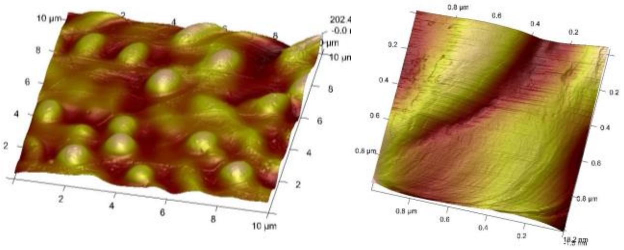

Figure S2. AFM pictures of T3 with histamine. left) Area of 10µm^2 right) Area of 1µm^2.

- **T5**: The surface of T5 is not uniform. The picture of 1 µm^2 shows a high roughness.

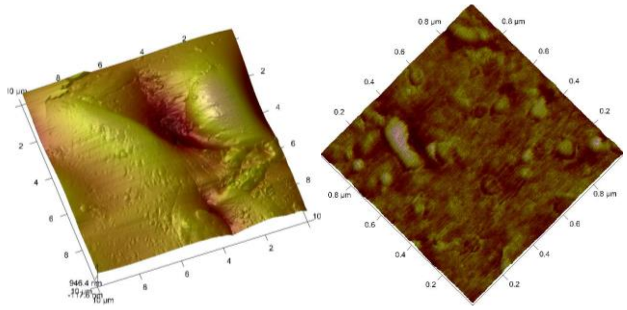

Figure S3. AFM pictures of T5 without histamine. left) Area of 10µm^2 right) Area of 1µm^2.

3.3. Fourier Transform Infra-Red spectroscopy (FTIR)

FTIR measurements were performed with a Nicolet Nexus FT-IR spectrometer equipped with an ATR Diamant Golden Gate. For the studied membranes we can identify the following vibrational bands assigned in the FTIR spectrum of **T2H:**

- C=C aromatic: usually between 1450 and 1600 cm^{-1} but, here, additional conjugation with C=N so slightly shifted towards lower wave numbers.
- C=N: 1640-1690 cm^{-1}
- C-O-C: between 1070 and 1150 cm^{-1}
- C-H alkanes: *bending: 1350-1480 cm^{-1}
  *stretch: 2850-3000 cm^{-1}:
- C-N: *aryl: 1250-1360 cm^{-1}
  *alkyl: 1025-1200 cm^{-1}

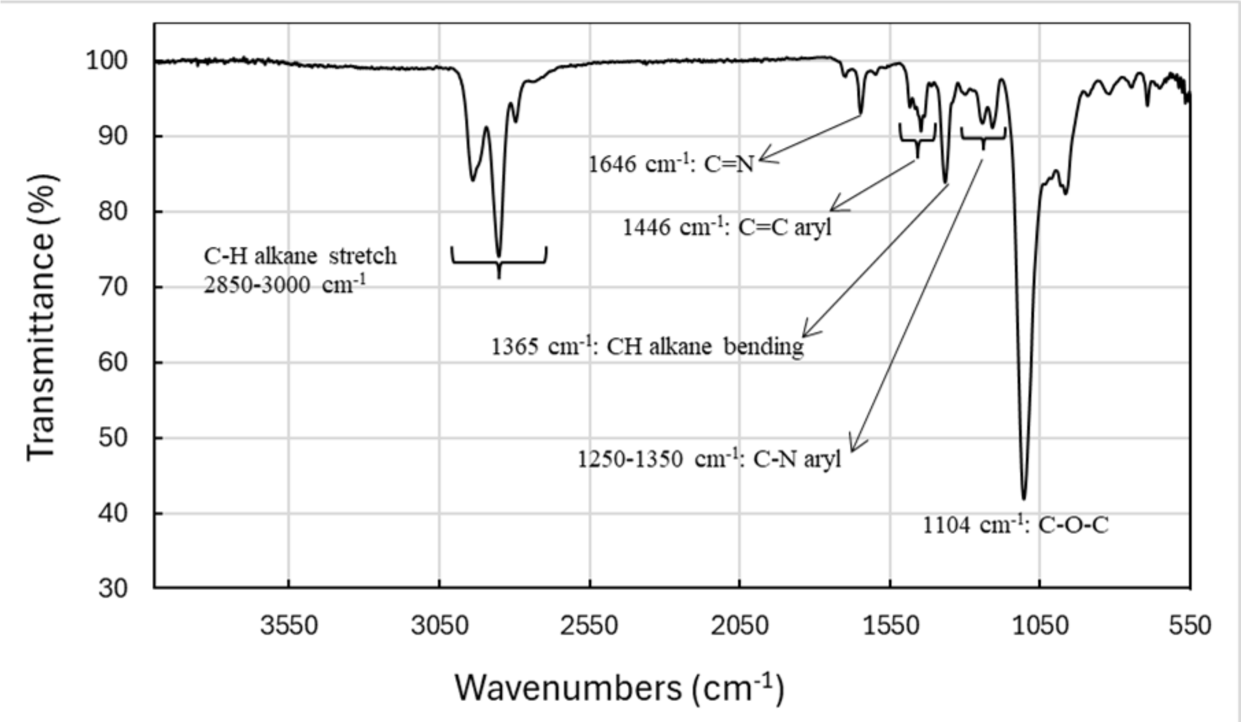

Figure S4. FTIR spectrum of T2H and assignment of main vibrational bands.

Table S3. Intensities of the main peaks observed in FTIR spectra of T1H-T6H and T1-T6.

Peak	Wave number (cm^{-1})	Peak intensity (% transmittance)											
		T1H	T2H	T3H	T4H	T5H	T6H	T1	T2	T3	T4	T5	T6
C-H strech 1	~2900	81.1	84.2	85.1	88.2	86.2	86.4	85.0	83.8	89.4	87.0	87.4	87.1
C-H strech 2	~2850	72.8	74.0	83.7	87.0	83.1	85.2	75.9	73.9	88.3	83.6	84.8	86.4
C=N	~1650	79.0	93.1	89.5	85.5	79.6	-	79.9	91.4	98.0	78.4	82.0	94.7
C=C aryl	~1450	82.1	90.7	85.8	85.6	84.4	89.6	89.2	89.4	93.0	84.5	87.6	89.9
C-H bending	~1350	78.1	83.9	76.2	85.8	78.0	77.6	82.7	82.1	92.7	80.6	78.8	90.5
C-O-C	~1100	39.6	41.8	38.5	64.6	44.2	36.3	40.4	36.6	41.1	43.4	42.2	41.1

Table S4. Intensities of the main peaks observed in FTIR spectra of T1H-T6H and T1-T6 based on aryl aromatic vibration around 1450 cm^{-1}, used as reference.

Peak	Wave number (cm^{-1})	Peak relative intensity compared to C=C aryl intensity											
		T1H	T2H	T3H	T4H	T5H	T6H	T1	T2	T3	T4	T5	T6
C-H strech 1	~2900	1.06	1.70	1.05	0.82	0.89	1.31	1.39	1.53	1.51	0.84	1.02	1.27
C-H strech 2	~2850	1.53	2.79	1.15	0.90	1.08	1.43	2.24	2.45	1.67	1.06	1.23	1.35
C=N	~1650	1.18	0.74	0.74	1.00	1.31	-	1.87	0.81	0.29	1.40	1.45	0.53
C=C aryl	~1450	1.00	1.00	1.00	1.00	1.00	1.00	1.00	1.00	1.00	1.00	1.00	1.00
C-H bending	~1350	1.22	1.73	1.68	0.98	1.41	2.16	1.60	1.68	1.04	1.25	The	0.94
C-O-C	~1100	3.39	6.25	4.34	2.46	3.58	6.14	5.53	5.96	8.39	3.65	4.66	5.84

❖ **T1H – T6H:** From **Table S4**, it can be seen that **T2H, T4H** and **T6H** has approximatively twice more –C-O- C- groups and more C-H groups than **T1H, T3H** and **T5H** respectively. This is coherent with their molecular formula: T2H is made from polyTHF 1100 (six –O-C_4H_8- groups) whereas **T1H** is made from polyTHF 350 (three –O-C_4H_8- groups). T3H and T4H have the same structure with different lengths of the PPO chains in the connector which is modified: there are 17 groups in each branch of **T3H** whereas only 2 for each branch of **T4H**. The relative intensity of –C-O-C- bonds around 1450 cm^{-1} is almost twice as high for T6H than for T5H. The T6 ether chain is made of 32 groups whereas T5 is made of 5 groups. Moreover, the difference for imine groups can be noticed with a more cross-linked feature for **T1H**, **T3H** and **T5H**. For all spectra it can be observed that there is a broad peak over 3000 cm^{-1}, related to water sorbed by the polymer. For all spectra the baseline is not flat; due to difficulties to achieve a good contact between the sample and the diamond tip of IR instrument when samples are glassy polymers.

❖ The same differences of the spectral behaviors exist between the membranes **T1-T6**. However, it can be noted that T3 (17 PPO groups) shows much more intense bands assigned to polyether chains, as C-O-C or C-H compared to T4 (2 PPO). The same tendency can be observed for the other couples of membranes (T1/T2, T5/T6) but with a less important difference in the ether content.

3.1. Swelling experiments

Table S5. Water sorption by weight for the different membranes.

Membrane	Amount of water sorbed (g) per volume of polymer (cm^3)	Uptake of water (cm^3_{water} / $cm^3_{polymer}$)
**T1H**	0.76	0.95
**T2H**	0.37	0.46
**T3H**	0.45	0.56
**T5H**	0.45	0.56
**T6H**	0.25	0.31
**T1**	0.20	0.25
**T2**	0.18	0.22
**T3**	0.10	0.12
**T4**	0.14	0.17
**T5**	0.16	0.20
**T6**	0.07	0.08

The membranes were weighted again after 2, 4, 6, 24, 28 and96 hours. For each point, three weight measurements were taken and an average value has been used for graphs plotting and calculations (Table S5). The weight increase related to the volume of the polymer sample has been calculated to compare the swelling of the membranes.

$$weight\ increase = \frac{Weight_{saturation} - Weight_{dry}}{Volume} \qquad (S1)$$

where the volume of the membrane is calculated from the area and the thickness of the membrane.

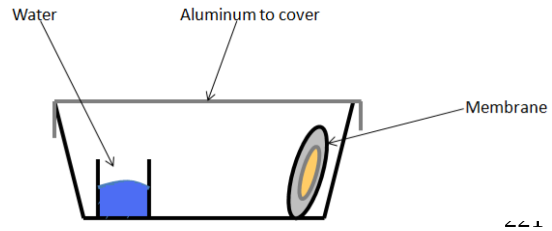

Figure S5. Schematic representation of the lab setup used for swelling experiments.

The membranes **T1H-T6H** containing hydrophilic histamine require longer equilibrium time to reach steady state (100h) and absorbs almost 3-4 times more water than the less hydrophilic membranes **T1-T6** (60 h). The main discussion relating to the membrane structure and water uptake is presented in the main paper.

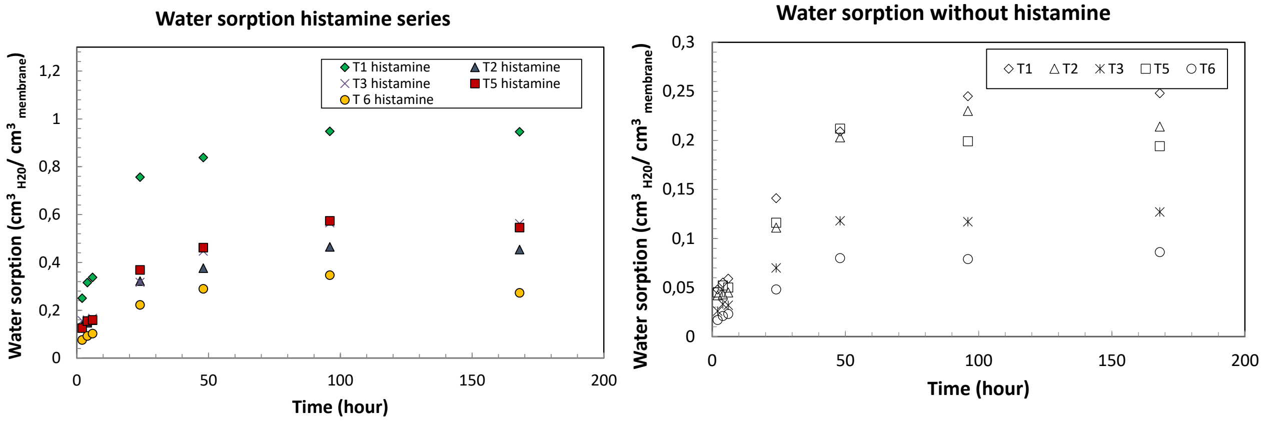

Figure S6. Uptake of water with time for T1H-T6H and T1-T6 membranes.

3.4. Gas permeation experiments

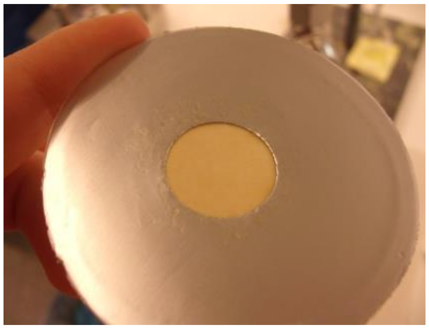

Figure S7. Preparation of the self-standing membrane for gas permeation testing.

3.4.1. **Single gas permeation experiments**

This experiment gives us the permeate side pressure as a function of the time. When the steady state is attained, this evolution is linear and the slope of the line (dp/dt) allows t calculate the gas permeability:

$$P_i = \frac{dp}{dt} * \frac{T^0}{P^0} * \frac{V_p}{T_p} * \frac{l}{P_f} * \frac{1}{A_m} \text{ (S2)}$$

Where T^0 (K) and P^0 (atm) are the standard temperature and pressure, V_p (cm^3) and T_p (K) are the volume and the temperature of the permeate side, l (cm) is the thickness of the membrane, P_f (K) is the pressure of the feed side and A_m (cm^2) is the area of the membrane.

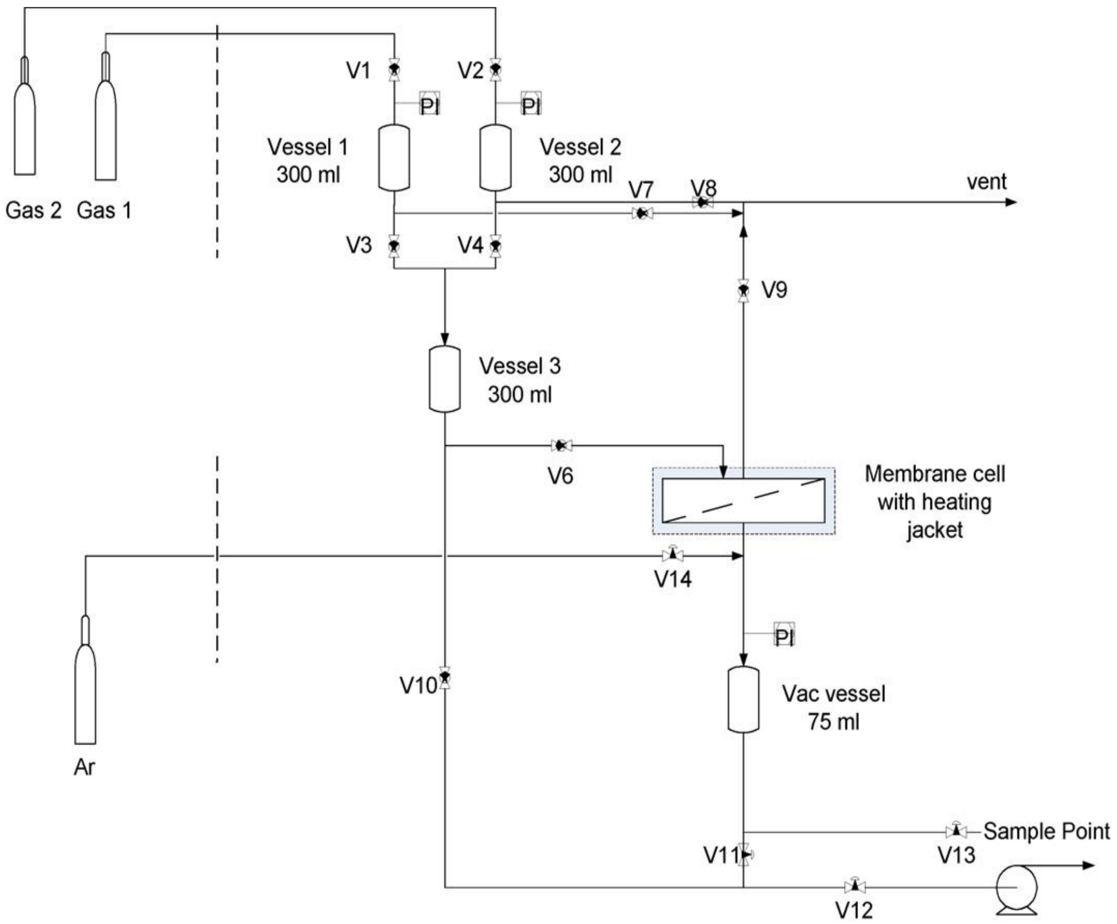

Figure S8. Gas permeation system flowsheet.

Table S6. Permeability and selectivity versus feed pressure

Membrane	Feed Pressure	Permeability (Barrer)			Selectivity	
		CO_2	N_2	CH_4	CO_2/N_2	CO_2/CH_4
**T1H**	3	2,28	0,12	0,12	19	19
	5	2,34	0,09	0,09	26	14
	8	2,52	0,11	0,11	23	14
	10	2,4	0,08	0,08	30	14
**T3H**	3	524	16	61	33	9
	5	424	15	61	28	7
	8		17	62	-	-
	10		17	65	-	-
**T5H**	3	31	0,76	2,12	41	15
	5	34	0,81	2,24	42	15
	8	40	0,8	2,24	50	18
	10	44	0,81	2,33	54	19

The recorded single gas permeabilities and calculated selectivity (ratio of permeabilities) and the main discussion relating the membrane structure to the permeation results is presented in the main paper. Additional data is presented here showing permeability variation with pressure for three of the membranes. T3H was damaged after 5 bar. The T1H and T5H show relatively constant gas permeability with pressure, consistent with a solution diffusion mechanism for gas transport, T5H showed compared with the other a slight increase in both CO_2 permeability and selectivity with pressure.

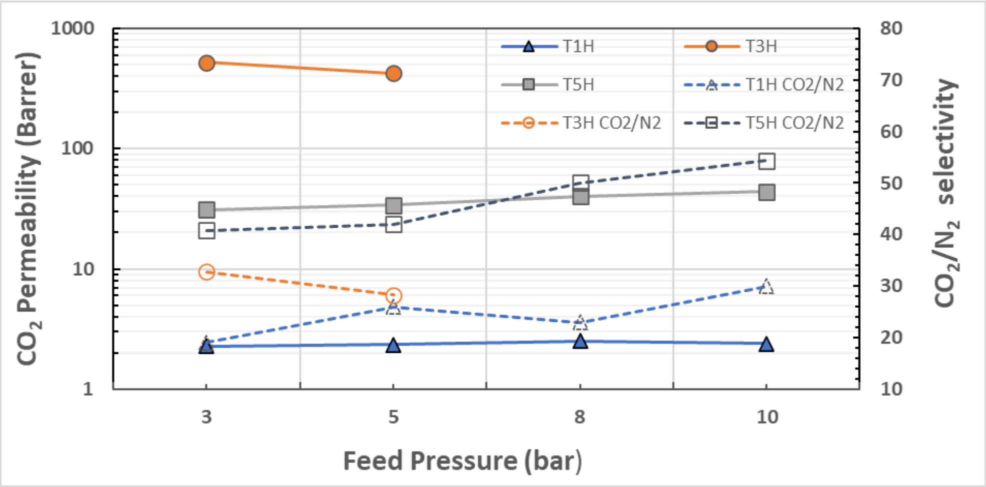

Figure S9. Effect of feed pressure at 25^{0}C for single gas permeation.

3.4.2. **Mixed gases permeation experiments**

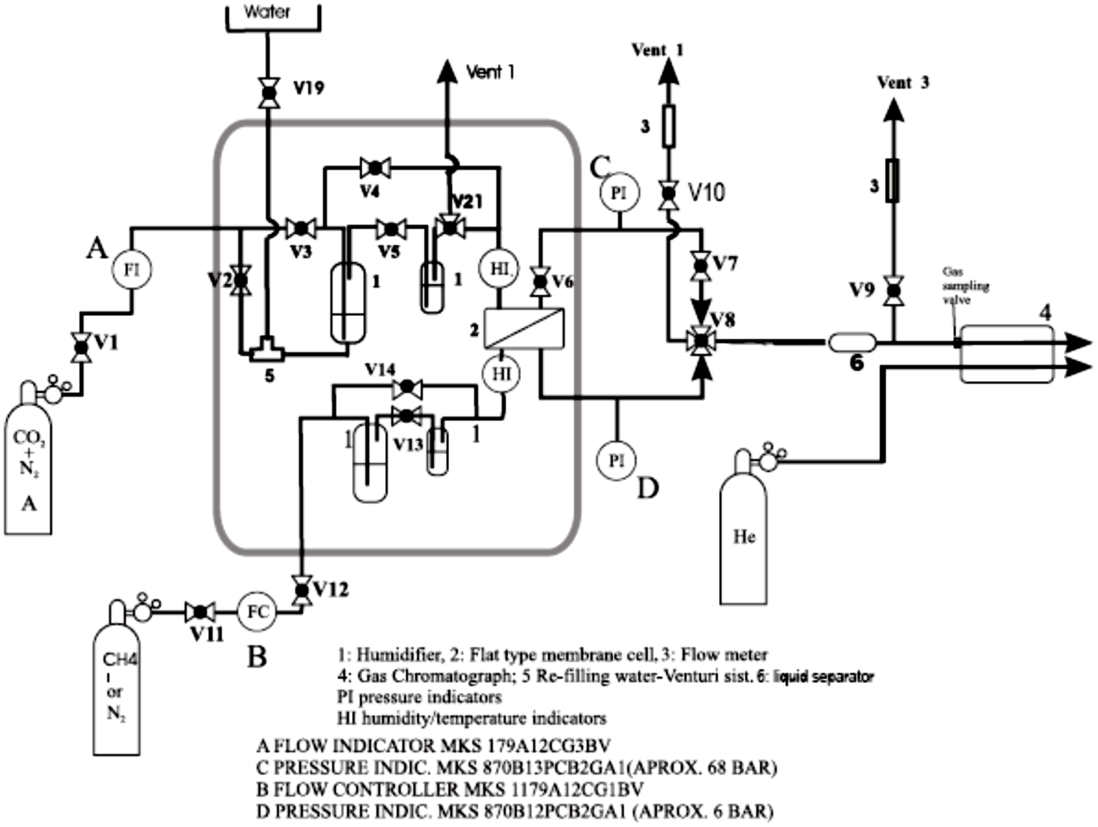

Figure S10. Schematic representation of the mixed gas permeation experiment set-up.

From the experiments, the amounts of nitrogen and carbon dioxide in the permeate gas and the flow rate are obtained. From those values and knowing the feed pressure and feed composition, it is possible to calculate the CO_2 and N_2 fluxes as well as the selectivities. The selectivity can be obtained from the composition of the permeate compared to the composition of the feed:

$$\alpha_{\frac{CO_2}{N_2}} = \frac{\left[\frac{x_{CO_2}}{x_{N_2}}\right]^{permeate}}{\left[\frac{x_{CO_2}}{x_{N_2}}\right]^{feed}} \text{ (S3)}$$

The flux of CO_2 –and the flux of N_2 by the same way- can be calculated with the following equation:

$$CO_2\ flux = \frac{Permeate\ flow * x_{CO_2}^{permeate}}{Area * \left(P_{feed} * x_{CO_2}^{feed} - P_{sweep} * x_{CO_2}^{permeate}\right)} \text{ (S4)}$$

The selectivity can also be calculated from the fluxes:

$$\alpha_{\frac{CO_2}{N_2}} = \frac{CO_2 flux}{N_2 flux} \text{ (S5)}$$

3.5. Gas sorption experiments.

The plot of the weight measurement versus the density of the gas gives a straight line whose slope gives the volume of the holder and at the intercept the weight of the sample holder. The density is calculated using the ideal gas law:

$$\rho = \frac{p * M}{R * T} \text{ (S6)}$$

where ρ is the density, p the pressure, M the molar mass of Helium, R the ideal gas constant and T the temperature.

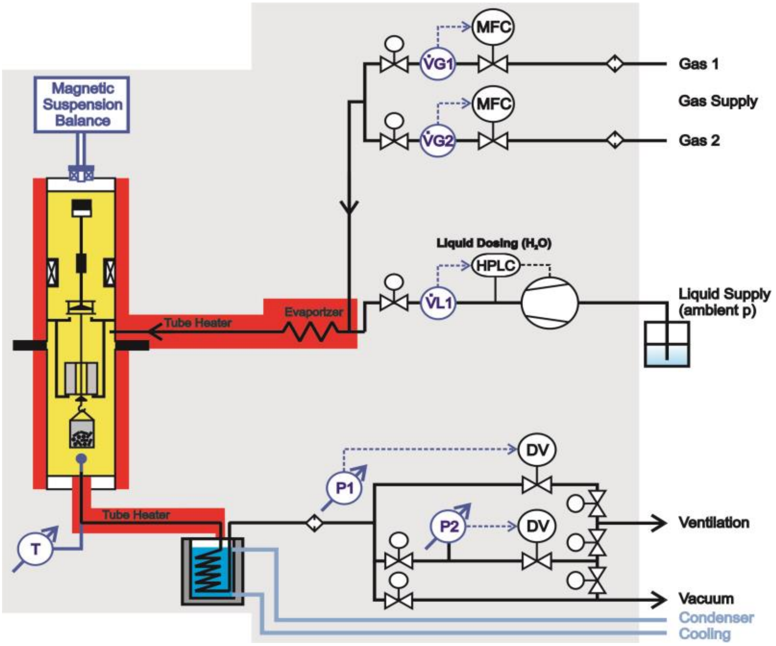

Figure S11. Magnetic suspension balance: schematic of the system (from Rubotherm manual).

It is also necessary to determine the weight and the volume of the sample. The sample is placed in the holder and the is subjected to different pressures of Helium. The plot of weight against density gives the volume and the weight of the sample. From the first blank measurement without sample and this one, it is possible to determine the weight and the volume of the sample. Before each sorption experiment, it is necessary to evacuate the entire system at least overnight to ensure complete desorption of gas from the sample and from all the instrument piping walls.

The weight of the sample and the holder needs to be corrected due to the buoyancy effect. The buoyancy is determined from the density of the gas (obtained from the pressure and the ideal gas law) and the volume of the sample and the holder (V_{sc+s}):

$$B = V_{sc+s} * \rho \text{ (S7)}$$

This buoyancy will be added to the weight given by the apparatus to obtain the corrected sample weight. If the initial weight of the sample with the holder is then subtracted, the weight of adsorbed gas is determined. The specific uptake is defined as the mass of adsorbed gas per gram of membrane:

$$specific\ uptake = \frac{m_{gas\ ads}}{m_s}\ \ expressed\ in\ \left[\frac{g_{gas\ ads}}{g_{membrane}}\right] \text{ (S8)}$$

This specific uptake can then be plotted against the pressure. The gases studied are non-ideal gases and deviations from ideality can occur. To take these deviations into consideration, fugacity was used and was calculated from the Soave-Redlich-Kwong (SRK) equation of state:

$$p = \frac{RT}{v-b} - \frac{a}{v(v+b)} \text{ (S9)}$$

where v is the molar volume, a and b are the model parameters. These parameters are characteristic for a component and are calculated from the following equations:

$$m = 0{,}48 + 1{,}574\omega - 0{,}176\omega^2 \text{ (S10)}$$

$$a = \frac{0{,}42747R^2T_c^2}{p_c}\left[1 + m\left(1 - T_r^{\frac{1}{2}}\right)\right]^2 \text{ (S11)}$$

$$b = \frac{0{,}08664RT_c}{p_c} \text{ (S12)}$$

T_r is the reduced temperature (T_r=T/T_c) and T_c and P_c are the critical temperature and pressure; w is the acentric factor of the component.
This equation is a cubic equation of v which means that the molar volume is the solution of a third-degree equation:

$$pv^3 - RTv^2 + (RTb + a - pb^2)v - ab = 0 \text{ (13)}$$

By solving this equation, the molar volume is obtained. This volume enables to calculate Z, the compressibility factor:

$$Z = \frac{pv}{RT} = \frac{v}{v-b} - \frac{a}{RT(v+b)}. \text{ (S14)}$$

The fugacity can then be calculated:

$$\ln\left(\frac{f}{p}\right) = -\ln\left(\frac{v-b}{v}\right) - \frac{a}{bRT}\ln\left(\frac{v+b}{v}\right) + Z - 1 - lnZ \text{ (S15)}$$

The volume measurement was always conducted according to the following procedure: 17 hours of evacuation at 60°C, 20h of evacuation at 25°C and then 10 segments at different pressures (0.5, 1, 1.5, 3, 5, 7, 10, 12, 15 and 20 bar). The volume measurement was followed by carbon dioxide sorption: 17h of evacuation at 60°C, 20h of evacuation at 25°C and then 8 segments at different pressures (0.5, 1, 1.5, 3, 5, 10, 15 and 20 bar). Desorption was then investigated by decreasing the pressure following the same steps. Each absorption and desorption segment had a duration of 3 hours of exposure to gas at a given pressure. After each segment, a static measurement was performed. All the valves of the instrument were closed for 30 minutes. The sorption calculations were investigated both in the flow mode and static mode. It was concluded that the results were more easily exploited in static mode measurement. The values used in calculation are an average of the 60 last data points from the static measurement at steady state/ equilibrium.

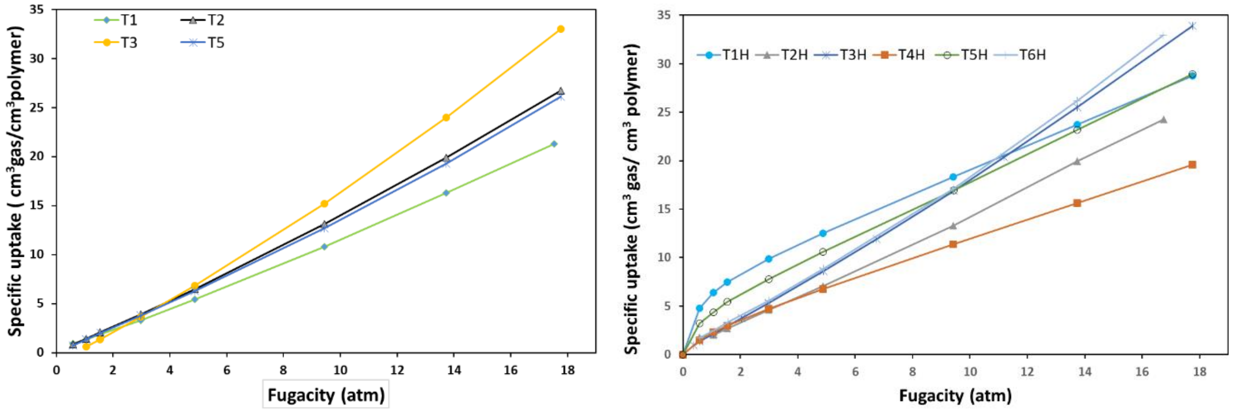

Figure S12. Left histamine and right without histamine series CO_2 sorption at 25 °C.

The CO_2 solubilities have similar values for all the membranes and the differences are more likely related to differences in polymer structure and not to the presence or absence of histamine functional groups. This trend is different when compared with water sorption trend from **Table S5** - the histamine series membranes are absorbing much more water than membranes without histamine.
The membranes from **T1 - T5** series do not show a hysteresis phenomenon and both sorption and desorption- follows Henry's law (**Figure S12**). This is consistent with the fact that the membranes are elastic/ rubbery. When histamine is present in the polymer network, for membranes ***T1H- T6H***, having more rigid structures than **T1 - T5**, both dual mode sorption model (DMS) (**T1H**, **T4H** and **T5H**) and Henry's law (**T2H**, **T3H** and **T6H**) had to be used to fit the experimental data. For most of these membranes except **T3H** and **T6H**, a hysteresis was observed and the difference between absorption and desorption is especially large for the more rigid structures on which DMS model was used (**Figure S13**).

The hysteresis is the result of the induction of new free volume sites during the sorption. The collapse of these new sites occurs in longer scale time than desorption of CO_2, a higher free volume exists during desorption and hysteresis can be observed.

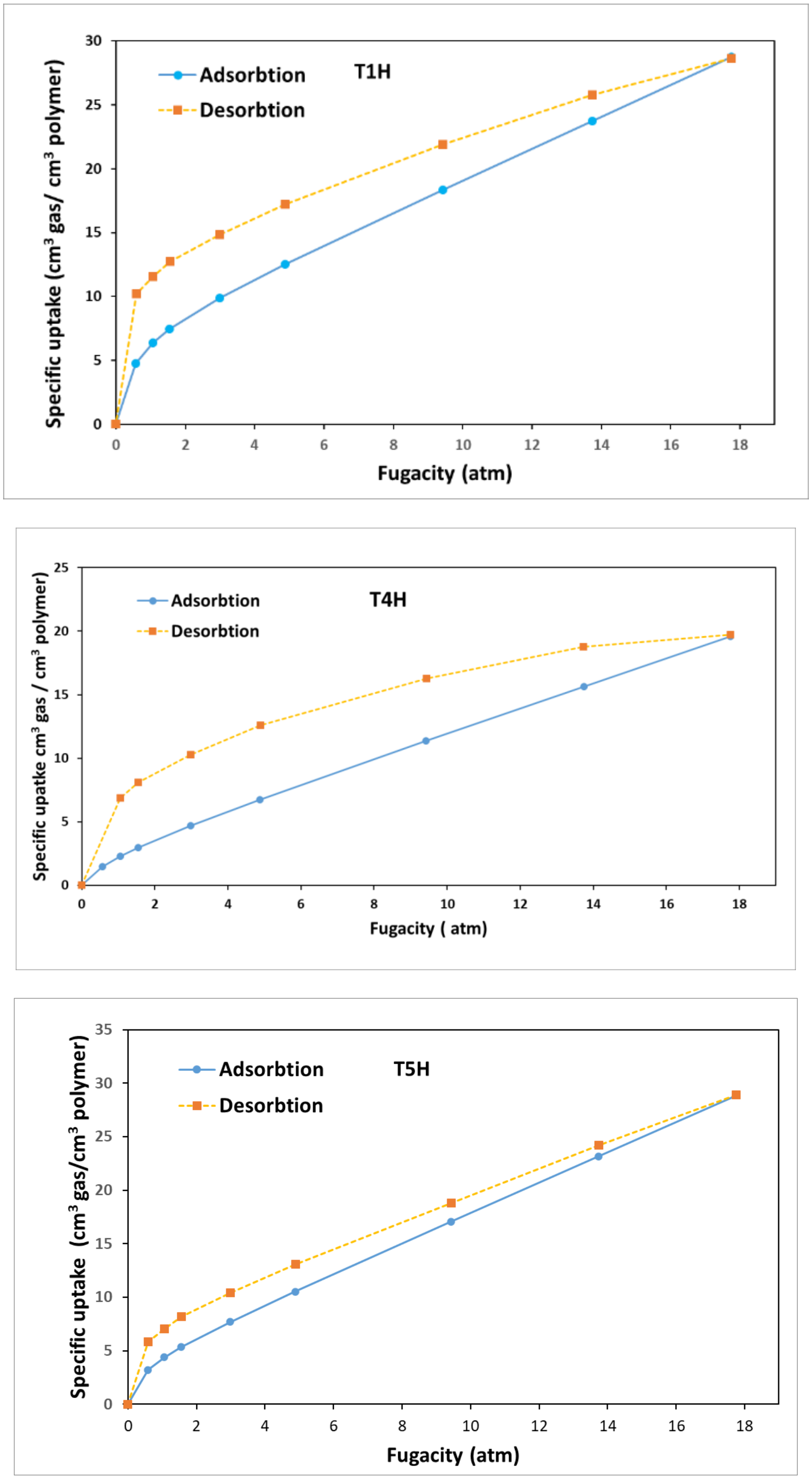

Figure S13. Hysteresis CO_2 sorption/desorption for the histamine series following DMS model.

Table S7 presents the results obtained by single gas sorption at 25 °C and pressure from 0.5 to 20 bar. The diffusion coefficient is calculated from the single gas permeabilities available and measured at 5 bar and 25 °C .

In the histamine series, the glassy membranes T1H, T4H and T5H present sorption according to dual mode sorption (DMS) model and have one -two order of magnitude smaller CO_2 diffusion coefficients compared with the rubbery **T2H** and **T3H** and **T6H** on which follow Henry's law. If solubility values are rather similar and comparable with values reported in literature for similar polymers, it can be noted that rubbery membranes have slightly bigger sorption values compared to the glassy membranes, but the difference is not important. In the series without histamine, all membranes except **T4** (not tested) are rubbery and follow Henry's sorption model.

Table S7. Sorption results. The diffusion coefficient is calculated from single gas CO_2 permeability at 5 bar, 25 °C.

Membrane	Solubility coefficient ($cm^3{}_{STP}$ /(cm^3 * atm))	D (cm^2/s) x 10^6	C'h ($cm^3{}_{STP}/cm^3$)	b (1/atm)	Comments
**with histamine**					
**T1H**	1.23	0.01	7.06	2.41	Dual sorption
**T2 H**	1.45	1.03			Henry's
**T3H**	1.86	1.74			Henry's
**T4H**	0.98		2.34	1.09	Dual sorption
**T5H**	1.41	0.18	3.97	2.60	Dual sorption
**T6H**	1.92	2.05			Henry's
**without histamine**					
**T1**	1.19	0.38	-	-	Henry's law
**T2**	1.46	1.30	-	-	Henry's law
**T3**	1.76	3.08	-	-	Henry's law
**T5**	1.42	0.18	-	-	Henry's law
**literature data**					
**PSF-NH_2**	0.4	-	21	0.26	Dual sorption
80PTMEO/PA12	0.96	1.74			
57PEO/PA6	0.88	0.57			
Amorphous PEO	1.3	0.25			

Despite following same sorption model and having relatively similar gas solubilities, a notable, one to two order of magnitude difference, can be observed between T1, T5 (low diffusion) and T2, T3 (high diffusion). If for T5 this effect can probably be attributed to a relatively high Tg = -14 ^{0}C (see **Table S2**), this difference in diffusivity is correlated with a difference in structure. The only common denominator, as in the case of single gas permeation results, seems to be length of polymer (PEO or PPO) chains, longer polymer chains giving higher diffusion regardless of the shape (star T3 or linear T1, T5, T6) or the blocks PPO -T3 and T5 and PEO T1 and T2. The combination of star shape with long PPO chains seems to be beneficial as it provides the higher CO_2 diffusion coefficient. Overall, it can be concluded that diffusivity has the biggest contribution to CO_2 permeability differences with the most rubbery membranes T3H, T6H and T2 and T3 giving the highest permeability along the T6 (not tested for sorption).

4. Atomistic simulations.

4.1 Sorption simulations

Sorption simulations were carried out to calculate single components and binary mixture sorption isotherms using the procedure implemented in a recent work on CO_2 sorption in polyimides (*45*). The methodology is based on sorption relaxation cycles (SRCs) that involve GCMC simulations integrated with *NPT*- and *NVT*-ensemble MD simulations. As the CO_2 pressure increases, ROFs swell with increasing CO_2 concentration, which increases its sorption capacity, until the CO_2 concentration reaches its equilibrium value at that pressure. GCMC runs were performed with 500,000 exchange moves, insertion and deletion combined, and with 10,000 rotational and translational moves. Consecutive 20 ps *NVT* and 20 ps *NPT* runs followed the GCMC step, in order to relax the polymer in the presence of CO_2 molecules. This cycle was repeated 50 times at each pressure to ensure that the equilibrium CO_2 concentration and polymer volume are reached. The MD simulations were performed using Nosé-Hoover thermostat and barostat with Velocity-Verlet algorithm. (*46, 47*) For the sorption of CH_4 and N_2 molecules, the *NVT* and *NPT* MD runs were set to 5 ns and the size of the simulation box were doubled in each of the three dimensions, to enhance statistics.

Once the sorption isotherms were calculated, they were fitted to the Henry's Law sorption model. The solubility, *S*, of the penetrant is defined as the concentration normalized by the feed pressure and is simply equal to the Henry's Law constant, *k*, for the rubbery polymers.

4.2 Diffusion simulations

Single-component self-diffusivities ($D_{s,i}$) of the permeants in the polymers were estimated from Einstein's relationship by evaluating the mean square displacement (MSD) from 20-ns long *NVT* MD runs via using Equation S16.

$$\mathrm{MSD(t)}=\frac{1}{N}\langle\sum_{N}|r_i(t)\text{-}r_i(0)|^2\rangle \quad \text{(S16)}$$

where r_i denotes the position vector of ith particle, *N* is the number of gas molecules and the angular brackets (⟨ ⟩), denote an ensemble average. The number of gas molecules was determined by calculating the average gas concentration at a given pressure via sorption simulations. Due to the statistical noise and non-linearity in the MSD behavior, the ensemble average in MSD was calculated using the time lag method (*48*), and its slope was used to estimate the diffusivity.

4.3 Estimation of permeabilities

In a rubbery membrane polymer, the permeability (P_i) of a gas "*i*" at a given penetrant feed pressure is expressed through the following equation according to the solution-diffusion definition:

$$P_i = S_i \times D_{s,i} = k_i \times D_{s,i} \quad \text{(S17)}$$

For the binary-gas diffusion simulations, prediction of permeabilities require evaluation of the Onsager coefficients. Open source OCTP tool for LAMMPS (*49)* was utilized to employ n^{th} order algorithm to obtain Onsager coefficients in this study (Table S8).

4.3.1.Glass transition temperatures

Figures S14, S15 show the variation of the specific volume (v) of the polymers as a function of temperature, evaluated through series of *NPT*-MD simulations. The temperature at which the slope of v vs T curve changes corresponds to the glass temperature of the polymer.

4.3.2. Permeability coefficients

For the binary-gas diffusion simulations, fluxes of individual species (J_i in the mixture can be expressed through Eqs. S17 and s18.

$$J_i = -D_{ii}\nabla c_i - D_{ij}\nabla c_j \qquad \text{(S18)}$$
$$J_j = -D_{ji}\nabla c_i - D_{jj}\nabla c_j \qquad \text{(S19)}$$

where, D_{ij} is the asymmetric mutual transport diffusivity ($D_{ij} \neq D_{ij}$). The fluxes can also be expressed in terms of the Onsager coefficients (L_{ij}) through Eqns. S20 and s21.

$$J_i = -L_{ii}\nabla \mu_i - L_{ij}\nabla \mu_j \qquad \text{(S20)}$$
$$J_j = -L_{ji}\nabla \mu_i - L_{jj}\nabla \mu_j \qquad \text{(S21}$$

with L_{ij} =L_{ji}. where μ_i is the chemical potential. The Onsager coefficients are evaluated by the following equation:

$$L_{ij} = \frac{1}{6N}\lim_{t\to\infty}\langle\sum_{l=1}^{N_i}(r_{li}(t) - r_{li}(0))\cdot\sum_{k=1}^{N_i}(r_{ki}(t) - r_{ki}(0))\rangle \qquad \text{(S22)}$$

Equation 3 can be expressed in the following form:

$$J_i = -\left[L_{ii}\left(\frac{\partial lnf_i}{\partial lnc_i}\right)_{T,c_j} + L_{ij}\left(\frac{\partial lnf_j}{\partial lnc_i}\right)_{T,c_j}\right]\nabla c_i - \left[L_{jj}\left(\frac{\partial lnf_i}{\partial lnc_j}\right)_{T,c_i} + L_{ij}\left(\frac{\partial lnf_j}{\partial lnc_j}\right)_{T,c_i}\right]\nabla c_j \qquad \text{(S23)}$$

where the derivative term is the thermodynamic factor given by the Darken's relation, which is equal to unity for the systems obeying Henry's law solubility regime. It follows then:

$$D_{ii} = L_{ii} + L_{ij} \qquad \text{(S24)}$$

$$D_{ij} = L_{jj} + L_{ij} \qquad \text{(S25)}$$

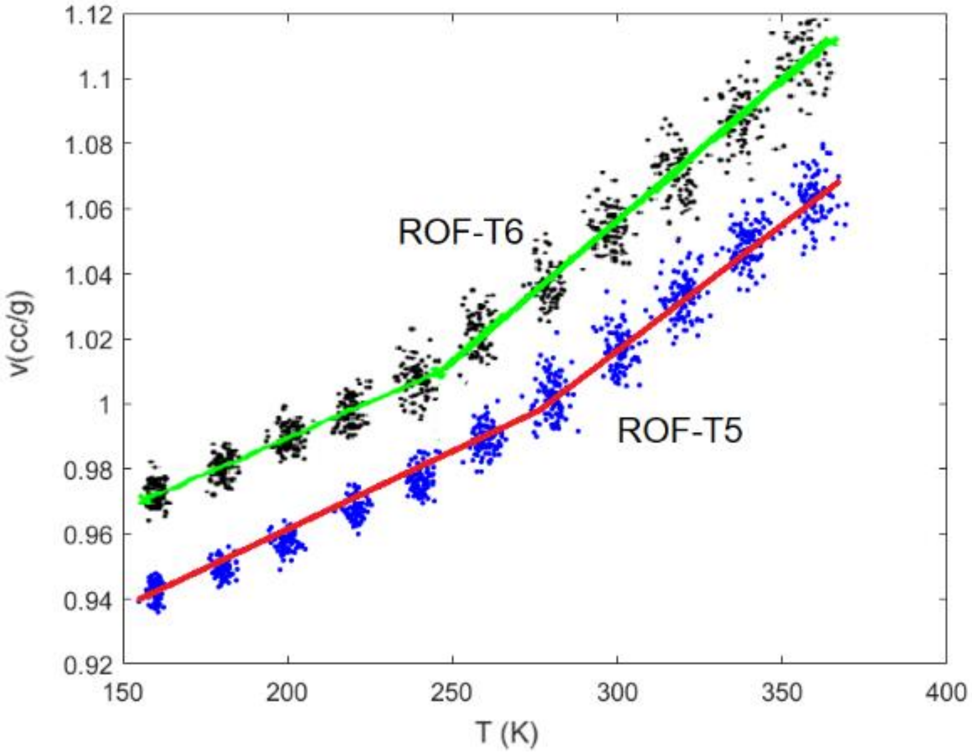

Figure S14. Variation of the specific volume with temperature for T5 and T6, where the inflection points of the slops correspond to the glass temperature of the polymer.

Table S8. Onsager coefficients for CO_2/N_2 (10:90) and CO_2/CH_4 (50:50) mixtures at 298 K and 5 bar.

	L_{ii} [10^{-6} cm^2/s]	L_{ij} [10^{-6} cm^2/s]		L_{ii} [10^{-6} cm^2/s]	L_{ij} [10^{-6} cm^2/s]
CO_2	4.78	0.144	CO_2	3.84	0.336
N_2	4.36	0.144	CH_4	2.26	0.336

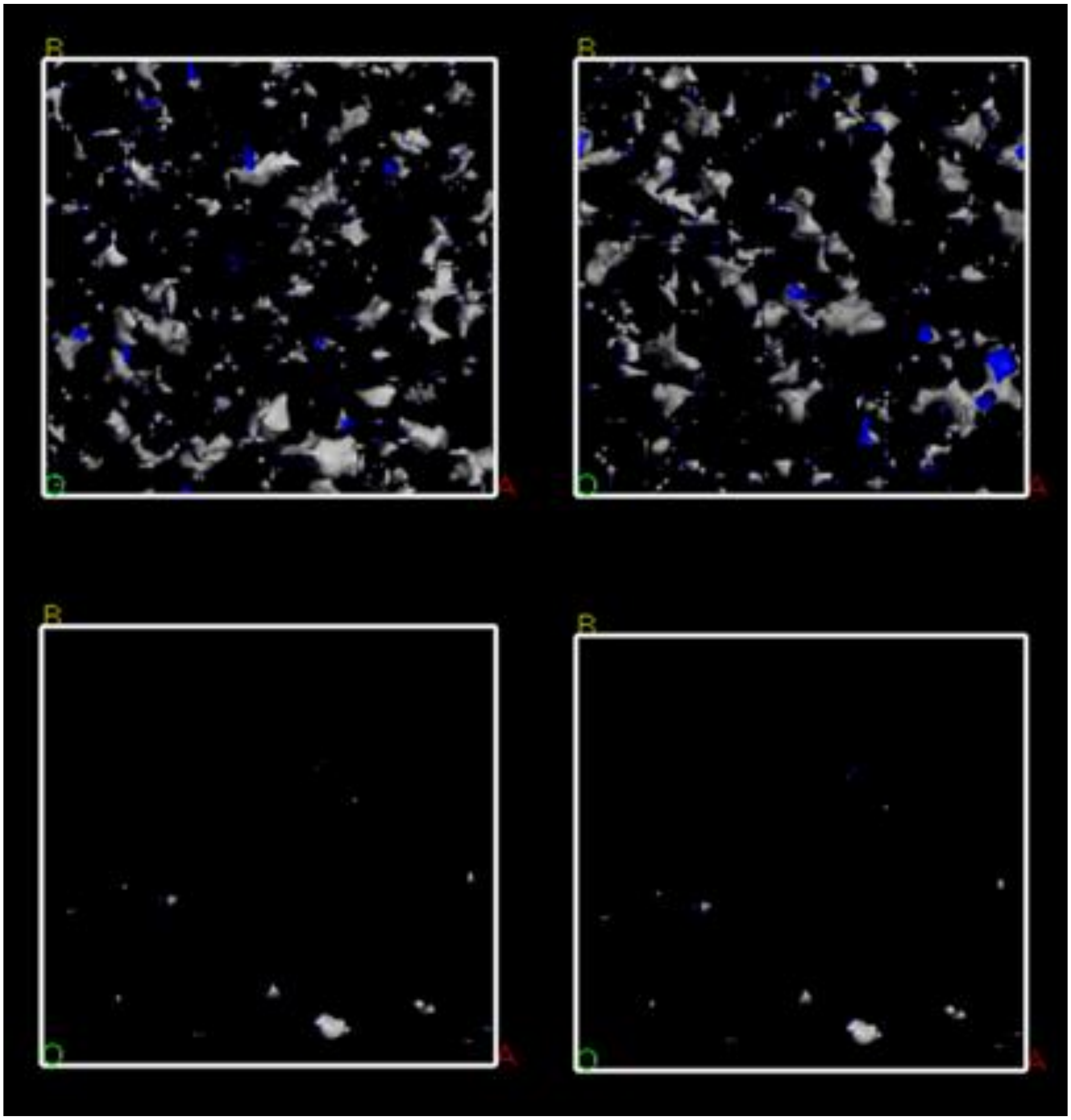

Figure S15. Distribution of free volume elements in T5 (left column) and T6 (right column) based on a probe radius of 1 Å (top row) and 1.65 Å (bottom row).

Data S1. Molecular simulation parameters for T5. This file contains the force field parameters used for each atom present in T5 alongside their partial charges.

Data S2: Molecular simulation parameters for T6. This file contains the force field parameters used for each atom present in T6 alongside their partial charges.

REFERENCES AND NOTES

1. S. A. Rackley, *Carbon Capture and Storage* (Elsevier Inc., 2010).

2. Y. Wang, L. Zhao, A. Otto, M. Robinus, D. Stolten, A review of post-combustion CO_2 capture technologies from coal-fired power plants. *Energy Procedia*, **114**, 650–665 (2017).

3. S. Sridhar, B. Smitha, T. M. Aminabhavi, Separation of carbon dioxide from natural gas mixtures through polymeric membranes—A review. *Sep. Pur. Rev.* **36**, 113–174 (2007).

4. C. A. Scholes, K. H. Smith, S. E. Kentish, G. W. Stevens, CO_2 capture from pre-combustion processes-strategies for membrane gas separation. *Int. J. Greenhouse Gas Control* **4**, 739–755 (2010).

5. H. Lin, B. D. Freeman, Materials selection guidelines for membranes that remove CO_2 from gas mixtures. *J. Mol. Struct.* **739**, 57–74 (2005).

6. T.-J. Kim, H. Vralstad, M.-B. Haag, Separation performance of PVAm composite membrane for CO_2 capture at various pH levels. *J. Membr. Sci.* **428**, 218–224 (2013).

7. M. Sandru, S. H. Haukebo, M.-B. Hagg, Composite hollow fiber membranes for CO_2 capture. *J. Membr. Sci.* **346**, 172–186 (2010).

8. M. Sandru, E. M. Sandru, W. F. Ingram, J. Deng, P. M. Stenstad, L. Deng, R. J. Spontak, An integrated materials approach to ultrapermeable and ultraselective CO_2 polymer membranes, *Science* **376**, 90–94 (2022).

9. P. M. Budd, N. B. McKeown, Highly permeable polymers for gas separation membranes. *Polym. Chem.* **1**, 63–68 (2010).

10. H. B. Park, C. J. Jung, Y. M. Lee, A. J. Hill, S. J. Pas, S. T. Mudie, E. Van Wagner, B. D. Freeman, D. J. Coockson, Polymers with cavities tuned for fast selective transport of small molecules and ions. *Science* **318**, 254–258 (2007).

11. M. Carta, R. Malpass-Evans, M. Croad, Y. Rogan, J. C. Janqsen, P. Bernardo, F. Bazzarelli, N. B. MeKeown, An efficient polymer molecular sieve for membrane gas separations. *Science* **339**, 303–307 (2013).

12. A. Pustovarenko, M. G. Goesten, S. Sachdeva, M. Shan, Z. Amghouz, Y. Alla Dikhtiarenko, T. Rodenas, D. Keskin, I. K. Voets, B. M. Weckhuysen, M. Eddaoudi, L. C. P. M. de Smet, E. J. R. Sudhölter, F. Kapteijn, B. Seoane, J. Gascon, Nanosheets of nonlayered aluminum metal-organic frameworks through a surfactant-assisted method. *Adv. Mater.* **30**, e1707234 (2018).

13. S. J. Datta , A. Mayoral, N. M. S. Bettahalli, P. M Bhatt , M. Karunakaran , I. D. Carja , D. Fan , P. G. M Mileo, R. Semino, G. Maurin, O. Terasaki, M. Eddaoudi, Rational design of mixed-matrix metal-organic framework membranes for molecular separations. *Science* **376**, 1080–1087 (2022).

14. S. Zhou, O. Shekhah, A. Ramírez, P. Lyu, E. Abou-Hamad, J. Jia, J. Li, P. M. Bhatt, Z. Huang, H. Jiang, T. Jin, G. Maurin, J. Gascon, M. Eddaoudi, Asymmetric pore windows in MOF membranes for natural gas valorization. *Nature* **606**, 706–712 (2022).

15. H. Fan, J. Gu, H. Meng, A. Knebel, J. Caro, High-flux membranes based on the covalent organic framework COF-LZU1 for selective dye separation by nanofiltration. *Angew. Chem. Int. Ed. Engl.* **57**, 4083–4087 (2018).

16. Y. Pan, B. Wang, Z. Lai, Synthesis of ceramic hollow fiber supported zeolitic imidazolate framework-8 (ZIF-8) membranes with high hydrogen permeability. *J. Membr. Sci.* **421–422**, 292–298 (2012).

17. T. C. Merkel, B. D. Freeman, R. J. Spontak, Z. He, I. Pinnau, P. Meakin, A. J. Hill, Ultrapermeable reverse-selective nanocomposite membranes. *Science* **296**, 519–522 (2002.

18. S. Friebe, L. Diestel, A. Knebel, A. Wollbrink, J. Caro, MOF-based mixed-matrix membranes in gas separation—Mystery and reality. *Chem. Ing. Tech.* **88**, 1788–1797 (2016).

19. S. Krause, N. Hosono, S. Kitagawa, Chemistry of soft porous crystals: Structural dynamics and gas adsorption properties. *Angew. Chem. Int. Ed. Engl.* **59**, 15325–15341 (2020).

20. R. Gaillac, P. Pullumbi, K. A. Beyer, K. W. Chapman, D. A. Keen, T. D. Bennett, F.-X. Coudert. Liquid metal–organic frameworks. *Nat. Mater.* **16**, 1149–1154 (2017).

21. N. Giri, M. G. Del Pópolo, G. Melaugh, R. L. Greenaway, K. Rätzke, T. Koschine, L. Pison, M. F. Costa Gomes, A. I. Cooper, S. L. James. Liquids with permanent porosity. *Nature* **527**, 216–220 (2015).

22. C. Kang, Z. Zhang, S. Kusaka, K. Negita, A. K. Usadi, D. C. Calabro, L. Saunders Baugh, Y. Wang, X. Zou, Z. Huang , R. Matsuda, D. Zhao, Covalent organic framework atropisomers with multiple gas-triggered structural flexibilities. *Nat. Mater.* **22**, 636–643 (2023).

23. T. D. Bennter, F.-X. Coudert, S. L. James, A. I. Cooper, The changing state of porous materials. *Nature Mater.* **20**, 1179–1187 (2021).

24. R. Dupuis, M. Barboiu, G. Maurin, Unravelling the pore network and gas dynamics in highly adaptive rubbery organic frameworks. *Chem. Sci.* **13**, 5141–5147 (2022).

25. G. Nasr, T. Macron, A.Gilles, C. Charmette, J. Sanchez, M. Barboiu, Metallodynameric membranes-are metallic ions facilitating the transport of CO_2? *Chem. Commun.* **48**, 11546–11548 (2012).

26. Y. Zhang, M. Barboiu, Constitutional dynamic materials– toward natural selection of function. *Chem. Rev.* **116**, 809–834 (2016).

27. G. Nasr, A. Gilles, T. Macron, C. Charmette, J. Sanchez, M. Barboiu, Tuning gas-diffusion through dynameric membranes: Toward Rubbery Organic Frameworks (ROFs). *Israel J. Chem.* **53**, 97–101 (2013).

28. W. Yave, A. Car, S. S. Funari, S. P. Nunes, K. V. Peinemann, CO_2-philic polymer membrane with extremely high separation performance. *Macromolecules* **43**, 326–333 (2010).

29. A. Car, C. Stropnik, W. Yave, K. V. Peinemann, Tailor-made polymeric membranes based on segmented block copolymers for CO_2 separation. *Adv. Funct. Mat.* **18**, 2815–2823 (2008).

30. S. R. Reijerkerk, A. Arun, R. J. Gaymans, K. Nijmeijer, M. Wessling, Tuning of mass transport properties of multi-block copolymers for CO_2 capture applications. *J. Membr. Sci.* **359**, 54–63 (2010).

31. I. Kocsis, I. M. Sorci, H. Vanselous, S. Murail, S. E. Sanders, E.Licsandru, Y.-M. Legrand, A. van der Lee, M. Baaden, P. B. Petersen, G. Belfort, M. Barboiu, Oriented chiral water wires in artificial transmembrane channels. *Sci. Adv.* **4**, eaao5603 (2018).

32. M. L. Williams, R. F. Landel, J. D. Ferry, The temperature dependence of relaxation mechanisms in amorphous polymers and other glass-forming liquids. *J. Am. Chem. Soc.* **77**, 3701–3707 (1955).

33. R. W. Baker, B. T. Low, Gas separation membrane materials: A perspective. *Macromolecules* **47**, 6999−7013 (2014).

34. K. Ghosal, R. T. Chern, B. D. Freeman, W. H. Daly, I. I. Negulescu, Effect of basic substituents on gas sorption and permeation in polysulfone. *Macromolecules* **29**, 4360–4369 (1996).

35. B. Comesana-Gandara, J. Chen, C. Grazia Bezzu, M. Carta, I. Rose, M.-C. Ferrari, E. Esposito, A. Fuoco, J C. Jansen, N. B. McKeown, Redefining the Robeson upper bounds for CO_2/CH_4 and CO_2/N_2 separations using a series of ultrapermeable benzotriptycene-based polymers of intrinsic microporosity. *Energ. Environ. Sci.* **12**, 2733–2740 (2019).

36. U. W. R. Siagian, A. Raksajati, N. F.Himma, K. Khoiruddin, I. G. Wenten, Membrane-based carbon capture technologies: Membrane gas separation vs. membrane contactor. *J. Nat. Gas Sci. Eng.* **67**, 172–195 (2019).

37. J. M. Kolle, M. Fayaz, A. Sayari, Understanding the effect of water on CO_2 adsorption, *Chem. Rev.*, **121**, 7280–7345 (2021).

38. V. I. Bondar, B. D. Freeman, I. Pinnau, Gas sorption and characterization of poly(ether-*b*-amide) segmented block copolymers. *J. Polym. Sci. Part B: Polym. Phys.* **37**, 2463–2475 (1999).

39. M. Prache, “Investigation of new polymeric membrances with controlled architecture for CO_2 capture,” thesis, Norwegian Uiversity of Science and Technology, Trondheim, Norway (2012).

40. M. G. Martin, J. I. Siepmann, Transferable potentials for phase equilibria. 1. United-atom description of *n*-alkanes. *J. Phys. Chem. B* **102**, 2569–2577 (1998).

41. J. Wang, R. M. Wolf, J. W. Caldwell, P. A. Kollman, D. A. Case, Development and testing of a general amber force field. *J. Comp.Chem.* **25**, 1157–1174 (2004).

42. D. Frenkel, B. Smith, *Understanding molecular simulation: From algorithms to applications* (Elsevier, 2001).

43. J. G. Harris, H. Y Kwong. Carbon dioxide's liquid-vapor coexistence curve and critical properties as predicted by a simple molecular model. *J. Phys. Chem.* **99**, 12021–12024 (1995).

44. J. J. Potoff, J. I. Siepmann, Vapor-liquid equilibria of mixtures containing alkanes, carbon dioxide and nitrogen. *AIChE J.* **47**, 1676–1682 (2001).

45. M. Balçık, S. Velioğlu, S. B. Tantekin-Ersolmaz, M. G. Ahunbay, Can crosslinking improve both CO_2 permeability and plasticization resistance in 6FDA–PBAPS/DABA copolyimides? *Polymer* **205**, 122789 (2020).

46. S. Nosé, A unified formulation of the constant temperature molecular dynamics methods. *J. Chem. Phys.* **81**, 511–519 (1984).

47. W. G. Hoover, Canonical dynamics: Equilibrium phase-space distributions. *Phys. Rev. A Gen. Phys.* **31**, 1695–1697 (1985).

48. T. Giorgino, Computing diffusion coefficients in macromolecular simulations: The diffusion coefficient tool for VMD. *J. Open Source Software* **4**, 1698 (2019).

49. S. H. Jamali, L. Wolff, T. M. Becker, M. de Groen, M. Ramdin, R. Hartkamp, A. Bardow, T. J. H. Vlugt, O. A. Moultos, OCTP*:* A tool for on-the-fly calculation of transport properties of fluids with the order-*n* algorithm in LAMMPS. *J. Chem. Inf. Model.* **59**, 1290–1294 (2019).
